# DNA Vaccines Against Mycoplasma Elicit Humoral Immune Responses in Ostriches

**DOI:** 10.3389/fimmu.2019.01061

**Published:** 2019-05-14

**Authors:** Martha Wium, Hester Isabella Jonker, Adriaan Jacobus Olivier, Dirk Uwe Bellstedt, Annelise Botes

**Affiliations:** ^1^Department of Biochemistry, Stellenbosch University, Stellenbosch, South Africa; ^2^Klein Karoo International, Oudtshoorn, South Africa

**Keywords:** ostrich, mycoplasma, DNA vaccine, OppA, antibody response

## Abstract

In ostriches, the population densities resulting from intensive rearing increases susceptibility to pathogens such as mycoplasmas. In addition to good management practices, vaccination offers an attractive alternative for controlling mycoplasma infections in food animals, instead of using antibiotics, which often leave unacceptable residues. The use of live attenuated vaccines, however, carry the concern of reversion to virulence or genetic recombination with field strains. Currently there are no commercially available vaccines against ostrich-infecting mycoplasmas and this study therefore set out to develop and evaluate the use of a DNA vaccine against mycoplasma infections in ostriches using an OppA protein as antigen. To this end, the *oppA* gene of “*Mycoplasma nasistruthionis* sp. nov.” str. Ms03 was cloned into two DNA vaccine expression vectors after codon correction by site-directed mutagenesis. Three-months-old ostriches were then vaccinated intramuscularly at different doses followed by a booster vaccination after 6 weeks. The ability of the DNA vaccines to elicit an anti-OppA antibody response was evaluated by ELISA using the recombinant OppA protein of Ms03 as coating antigen. A statistically significant anti-OppA antibody response could be detected after administration of a booster vaccination indicating that the OppA protein was successfully immunogenic. The responses were also both dose and vector dependent. In conclusion, the DNA vaccines were able to elicit an immune response in ostriches and can therefore be viewed as an option for the development of vaccines against mycoplasma infections.

## Introduction

The ostrich (*Struthio camelus* var. *domesticus*) is the largest living, flightless bird species belonging to the ratite group (1). Although ostriches are traditionally endemic to Africa, parts of Arabia and the Middle East (2), they are reared in several countries across the world. South Africa, however, is currently the largest producer of ostrich products (meat, leather, and feathers) on international markets (3). Here, the majority of ostriches destined for slaughter are reared in free-range systems, but when environmental conditions are unfavorable for grazing, they are reared in grow-out camps from about 3 months of age (4). *Mycoplasma*, a genus of bacteria that belongs to the prokaryotic class Mollicutes, is known to be highly infectious in these intensive rearing systems (5, 6). Characteristic features of mycoplasma include the lack of a cell wall, small AT-rich genome and a minimal set of genes (7, 8). The species found to specifically infect ostriches are “*Mycoplasma struthionis* sp. nov.” str. Ms01, *Mycoplasma* sp. str. Ms02, and “*Mycoplasma nasistruthionis* sp. nov.” str. Ms03 (9, 10). They are typically associated with upper respiratory tract infections (9), reduced growth rates, downgrading of carcasses and in extreme cases, chick mortalities (5, 9). The severity of infections increases with exposure to environmentally induced stress, other bacterial and viral infections. Of the three *Mycoplasma* species, “*Mycoplasma nasistruthionis* sp. nov.” str. Ms03 was found to have the highest prevalence (6) amongst ostriches and therefore was the focus of this study.

Mycoplasma infections are currently managed using a combination of biosecurity systems and antibiotics. With antibiotics there is the risk of resistance (11) as well as an accumulation of residues in meat with concomitant risks for consumers (12). Development of whole organism vaccines for use in ostriches is limited by the slow-growing nature of these organisms and the cost of medium required to sustain growth. DNA vaccines, on the other hand, do not require large scale cultivation of the pathogen, can be produced at relatively low cost and are more temperature stable than other vaccines (13, 14). It is therefore an attractive alternative for use in ostriches where the vaccine market is small, relative to e.g., poultry, and ostriches are usually farmed in semi-arid or arid regions where temperatures are frequently high and cold storage of vaccines can be problematic. Similar to live vaccines, they are also able to stimulate humoral and cellular immunity as indicated by measuring antibody, T-helper cell and cytotoxic T-lymphocyte responses (15). but unlike live vaccines cannot revert back to an infectious form (16). El Gazzar et al. (17) has reported the reversion to virulence of a commonly used live mycoplasma vaccine in poultry, ts-11, whilst another has reported possible genetic recombination between live vaccines and field strains upon long term use (18). This exchange or transfer of genes could have a negative impact on either the pathogenicity or transmissibility of field strains. Chromosomal integration of DNA vaccines is of some concern, but preclinical and clinical studies have shown the rate of integration of plasmid DNA into the host genome to be lower than that of spontaneous mutations (19, 20). To date, DNA vaccines have not been tested in ostriches or any other ratites. The aim of this study was to use the OppA protein of “*M. nasistruthionis* sp. nov.” str. Ms03 as antigen in the development and evaluation of DNA vaccines in ostriches at different doses and using different vectors.

A DNA vaccine is a plasmid expression vector containing a gene that codes for a protein antigen. As a result of their parasitic lifestyle (7), mycoplasmas possess, and rely on, a wide range of transmembrane transport systems for their survival. The extracellular components of these transporters are ideal targets for vaccine development (21, 22). In this study the extracellular OppA domain of an oligopeptide permease (Opp) transporter was chosen as protein antigen (23, 24). Using mouse models, OppA has to date been evaluated as antigen in subunit vaccines against *Brachyspira pilosicoli* (25), *Moraxella catarrhalis* (26), and *Yersinia pestis* (27), as well as against *Haemophilus parasuis* in pigs (28). OppA has, however, not been evaluated in any organism as part of a DNA vaccine. In this study we report, for the first time, that a DNA vaccine can elicit a humoral immune response in ostriches using OppA as antigen. This response was both dose and vector dependent.

## Materials and Methods

### DNA Vaccine Development

#### Site-Directed Mutagenesis of the oppA Gene

Cultures of “*M. nasistruthionis* sp. nov.” str. Ms03 (GenBank: KM410300.1) were obtained from Mr J.J. Gouws (Faculty of Veterinary Science, Onderstepoort, University of Pretoria). Genomic DNA (gDNA) was isolated from these cultures using a method described by Hempstead (29). The Type A *oppA* gene (24) was amplified from gDNA and cloned into the pGEM®-T Easy vector (Promega). Primers used are listed in Supplementary Table 1 and PCR conditions described in the Supplementary Procedures. To allow eukaryotic expression of the *oppA* gene, it was subjected to site-directed mutagenesis (SDM) to change 16 mycoplasma tryptophan codons (TGA) to universal tryptophan codons (TGG). The PCR primers used for SDM are listed in Supplementary Table 1 and the SDM procedure outlined in Supplementary Procedures. Correct mutation of the gene was confirmed with sequencing by the Central Analytical Facility, DNA Sequencing Unit of Stellenbosch University using an ABI® 3100 Genetic Analyser (Applied Biosystems, USA).

#### Preparation of DNA Vaccines

pCI-neo (Promega) and VR1020 (Vical Inc.) were chosen as vaccine vectors (Supplementary Figure 1). The pCI-neo vector is a mammalian expression vector that contains a CMV enhancer/promoter region, chimeric intron and SV40 late polyadenylation signal sequence. The VR1020 vector also contains a CMV enhancer/promoter region, but in combination with a CMV intron, tissue plasminogen activator (TPA) signal peptide sequence and a bovine growth hormone polyadenylation signal sequence. The mutated *oppA* gene was sub-cloned into each vector using restriction digestion. For pCI-neo, AccI and MluI (FastDigest, Thermo Scientific) and for VR1020, BamHI (Fermentas) restriction enzymes were used according to the manufacturer's instructions.

Cultures of the pCI-neo_*oppA* and VR1020_*oppA* vaccine plasmid were prepared and the respective plasmids purified with an Endotoxin-free plasmid DNA purification kit (NucleoBond® Xtra Midi plus EF, Macherey-Nagel, Germany). Yields were determined using a Nanodrop spectrophotometer. Prior to large scale plasmid isolation for vaccination, the NucleoBond kit's ability to yield supercoiled pCI-neo_*oppA* and VR1020_*oppA* plasmids was evaluated by digesting plasmids with the AccI and BamHI restriction enzymes, respectively, followed by electrophoresis on a 1% (w/v) agarose gel. To prepare a sufficient amount of plasmid for vaccination, the plasmids were prepared in batches over several days. The purified plasmids were again evaluated as before to confirm the integrity and supercoiling of the plasmids prior to being used for vaccination.

The isolated plasmids were diluted to 100, 600, and 1,200 μg/ml with sterile PBS (137 mM NaCl, 2.7 mM KCl, 10 mM Na_2_HPO_4_, and 1.5 mM KH_2_PO_4_, pH 7.2), respectively, a day before use. Dilutions were prepared under aseptic conditions, in sterile serum glass bottles, closed with inert silicone stoppers and sealed with a tear away center aluminum cap (Sigma-Aldrich).

### DNA Vaccine Trial

#### Ethical Approval

Ethical approval was obtained from the Stellenbosch University Animal Ethics Committee (SU-ACUM 13-00019) as well as the South African Department of Agriculture, Forestry and Fisheries (Reference: 12/11/1/1/3) in terms of section 20 of the Animal Disease Act 1984 (Act No. 35 of 1984).

#### Animals Used and Trial Location

A group of 140 ostrich chicks of ±3-months-old were randomly selected from a chick rearing unit in the Fraserburg district (Northern Cape Province, South Africa). Since Fraserburg is outside the major commercial ostrich production region, this limits pathogen exposure during the critical 3–4 months-old rearing phase (30). As per standard practice, upon reaching a weight of about 40 kg (±4 months of age), trial ostriches were transported back (±280 km) to a commercial ostrich farm in the Oudtshoorn district (Western Cape Province, South Africa). Three-month-old chicks were chosen for vaccination to allow sufficient time for a primary immune response to develop before being transported to a higher stress environment with concomitant increase in pathogen load and increased risk of exposure to mycoplasma.

The chicks were quarantined for 28 days after relocation and random testing was performed for avian influenza. This is required by avian influenza control measure before ostriches can be released and allowed to mix with other populations on any farm to which they have been transported.

At the start of the trial, each ostrich was tagged with a unique number for identification under the right wing, in accordance with standard ostrich farming practices. The trial was conducted under similar conditions to which a commercial vaccine would be administered and therefore trial ostriches were at all times housed and treated in the same manner as non-trial ostriches on the farms. In Fraserburg, the chick rearing units were open air camps (1,250 m^2^) with 120–150 chicks per camp. In Oudtshoorn, ostriches were kept in open air grow-out camps (25,000 m^2^) with 100–120 ostriches per camp. The ostriches received food and water *ad libitum* and were only handled by trained and experienced farm personnel. Trial birds were not kept separately but grouped with other ostriches of the same weight and age.

#### Vaccination Trial Design

The ostriches were randomly allocated to seven treatment groups of 20 ostriches each. The pCI-neo_*oppA* and VR1020_*oppA* vaccine groups were vaccinated at a dose of 100, 600, and 1,200 μg, respectively. The last group was the control group that did not receive any vaccine. The dose typically administered to poultry ranges from 0.25 to 800 μg when injected intramuscularly, with a booster dose after 2–3 weeks (31–38). Dunham (39) indicated that larger animals may require a larger dose of 500–2,500 μg, but that this needs to be optimized for individual vaccines since the dose required is influenced by the target species, its size and the efficiency of plasmid delivery. The different doses used in our study were therefore selected to compensate for the increase in weight of the ostriches during the course of the trial and the fact that no adjuvant was used in the vaccine formulation to limit possible skin reactions which can impact hide quality.

Vaccine doses were administered in a single 1 ml volume at week 0 and a booster injection administered at week 6 by intramuscular injection in the upper thigh. Blood samples (4 ml) were drawn from the jugular vein using 18Gx1/2 ″needles (Vacuette®) and collected in Vacuette® Z serum sep clot activator tubes at week 0, 3, 6, and 9. Serum was separated by centrifugation at low speed for 10 min and stored at −20°C. Week 0 and 3 samples were collected in Fraserburg, and week 6 and 9 samples in Oudtshoorn. The ostriches were moved from Fraserburg to Oudtshoorn 12 days prior to the week 6 sampling and booster injections were therefore administered during the quarantine period. Ostriches were monitored after vaccination for any adverse reactions.

### Evaluation of Humoral Immune Responses

#### Expression and Purification of the Recombinant OppA Protein

For analysis of anti-OppA antibody responses, recombinant OppA protein was produced for use as coating antigen in an enzyme-linked immunosorbent assay (ELISA). To this end, the SDM corrected *oppA* gene was PCR amplified and sub-cloned into a pGEX-4T-1 vector (GE Healthcare Life Science, UK) using restriction digestion. Primer sequences with BamHI and NotI restriction sites are shown in Supplementary Table 1.

For protein expression, the pGEX-4T-1_*oppA* plasmid was transformed into *Escherichia coli* BL21(DE3)pLysS cells (Promega) and freezer stocks prepared. An overnight culture (from a freezer stock) and expression cultures were prepared using Terrific-Broth (TB) medium. Expression of the glutathione S-transferase (GST) OppA fusion protein was induced at an OD_600_ between 0.4 and 0.6 by the addition of 0.4 mM IPTG, and cells harvested at 0 and 6 h by centrifugation (10,000 × g at 4°C). The cells were resuspended in 1xTen50 extraction buffer [10 mM Tris-HCl, 1 mM EDTA, 50 mM NaCl, 0.1% Triton X-100, 0.2 M dithiothreitol and 10% glycerol (v/v)] at 100 μl extraction buffer per 1 ml culture used for centrifugation. Protease inhibitor was also added to the Ten50 buffer (1 tablet per 10 ml extraction solution of complete ULTRA Tablets, Mini, EASYpack tablet, Roche).

The GST-OppA protein was isolated using glutathione-agarose chromatography (Sigma-Aldrich) under gravity flow at 4°C according to the manufacturer's instructions. A sample (8 ml) was prepared for loading of the column by treating the resuspended pellets with three freeze-thawing cycles (20 min at 37°C followed by 10 min at −80°C) and five cycles of 2 s sonication followed by 2 min on ice. The samples were then triturated three times through a 25Gx5/8″ needle (Avacare) into a 1 ml Injekt-F syringe (BBraun). This was repeated using a 23Gx1/4" needle (Nipro) followed by centrifugation at 10,000 × g for 10 min (4°C) to obtain a clear supernatant which was loaded onto the column under gravity flow at 4°C.

Fractions (1 ml) were collected and their protein concentration determined using a modified Bradford assay (40). Expression and isolation products were analyzed using SDS-PAGE (40) and Western blot analysis (41).

#### Evaluation of Anti-OppA Antibody Response

Microtiter plates (Maxisorp, Nunc) were coated overnight at 4°C with 100 μl of recombinant GST-OppA protein diluted to 10 μg/ml in carbonate buffer (50 mM NaHCO_3_, pH 9.6). To block non-specific binding, 200 μl/well casein buffer (10 mM Tris-HCl, 154 mM NaCl, 0.5% casein and 0.02% Thiomersal, pH 7.6) was added and the plate incubated for 1 h at 37°C. Serum samples were diluted 1:100 with casein-Tween [casein buffer containing 0.1% (v/v) Tween® 20] and 100 μl/well added to the plate in triplicate before incubation for 1 h at 37°C. Biotinylated rabbit anti-ostrich Ig polyclonal antibodies, prepared as previously described (42), were next added at a dilution of 1:100 in casein-Tween and incubated for 1 h at 37°C. A streptavidin horseradish peroxidase (HRP) conjugate mixture [2 ml Streptavidin HRP (Invitrogen), 38 ml 0.5% casein buffer, and 40 ml 50% glycerol], diluted 1:100 with casein-Tween, was then added (100 μl/well) and the plate incubated for 1 h at 37°C. Finally, 100 μl/well substrate solution (0.5 mg/ml ABTS, 0.5 μl/ml H_2_O_2_, 0.1 M citrate buffer, pH 5) was added and absorbance measured at 405 nm with a Thermo Scientific Multiskan EX plate reader after 30 min incubation at 37°C. After each incubation step the content of the plate was first decanted followed by washing five times with PBS-Tween [137 mM NaCl, 2.7 mM KCl, 10 mM Na_2_HPO_4_ and 1.5 mM KH_2_PO_4_, pH 7.2, 0.1% (v/v) Tween® 20] and washing three times with Milli-Q® water. Between the coating and blocking steps washing was not applied.

A first-row column blank (containing all components except ostrich serum) was used on each plate to blank absorbance values. All plates contained controls to monitor plate to plate variation. The controls were serum samples representing the week 0, 3, 6, and 9 sampling points of a single ostrich, randomly selected from the pCI-neo_*oppA* 1,200 μg group based on high titers produced after vaccination. Prior to the analysis of samples, the ELISA was optimized with regard to coating concentration (1–10 μg/ml), serum dilution (1:40–1:640) as well as number of washing cycles between steps. Non-specific binding of anti-OppA antibodies during ELISA analysis was evaluated by coating the wells with carbonate buffer (100 μl/well) containing no capture antigen and serum from a vaccinated bird that gave a high absorbance value at a dilution of 1:100 when using the GST-OppA protein as capture antigen.

The absorbance values obtained are referred to as ELISA titres, which are a measurement of the antibody levels produced when using a serum dilution of 1:100 in the ELISA.

### Monitoring Weight and Mycoplasma Infections

The weight of trial birds was monitored and recorded at weeks 0, 6, and 9. To determine the possible influence of existing mycoplasma infections on immune response data, trachea swabs were collected at weeks 0, 3, 6, and 9 and tested for the presence of mycoplasma infections by PCR. Birds were, however, not excluded from the trial if they tested positive since ostriches are often naturally infected with mycoplasmas. Swab samples were collected using dry sterile swabs (Copan) which were then rinsed in 200 μl sterile PBS buffer. The eluate was tested by PCR using a generic primer pair specific for the *Mycoplasma* genus (9). Positive samples were further evaluated for the presence of the ostrich-infecting mycoplasmas Ms01, Ms02, and Ms03 (9).

### Statistical Analysis of Trial Data

Statistical analysis of the ELISA titer data was performed using the General Linear Model (GLM) procedure in the Agrobase Generation II® (Agronomix Software Inc.) software. Analysis of variance (ANOVA) and least significant difference (LSD) values were calculated at a significance level of 0.05.

## Results

### DNA Vaccine Development

#### Site-Directed Mutagenesis of the OppA Gene

The 3867 bp *oppA* gene was successfully amplified from the Ms03 gDNA and cloned into a pGEM®-T Easy vector as confirmed by sequencing. Using SDM, all 16 mycoplasma TGA codons in this plasmid were successfully mutated to universal TGG codons in seven consecutive steps of SDM as confirmed by sequencing (Supplementary Figure 2).

#### Preparation of DNA Vaccines

The mutated *oppA* gene was successfully sub-cloned into the pCI-neo and VR1020 vaccine vector as confirmed by sequencing (Supplementary Figure 2). Large scale production of the DNA vaccines was achieved and approximately 89 mg of plasmid was isolated for each of pCI-neo_*oppA* and VR1020_*oppA*. The 260/280 absorbance ratio of the isolated plasmids ranged from 1.89 to 1.95. The integrity of the isolated plasmids was confirmed with agarose electrophoresis and most of the pDNA was in the required supercoiled conformation. A supercoiled conformation has a higher transfection rate into mammalian cells, is less susceptible to intracellular degradation and is more effective at inducing an immune response than other plasmid conformations (43, 44).

### DNA Vaccine Trial

Ostriches were vaccinated with the prepared DNA vaccines and no adverse reactions were observed at the injection sites (redness, swelling, inflammation, or allergic reaction) during and after the trial. Ostriches resumed normal behavior such as eating, walking around and exploring immediately after vaccination (no lameness or loss of appetite).

### Evaluation of Humoral Immune Responses

#### Expression and Purification of the Recombinant OppA Protein

**Figure 1:**
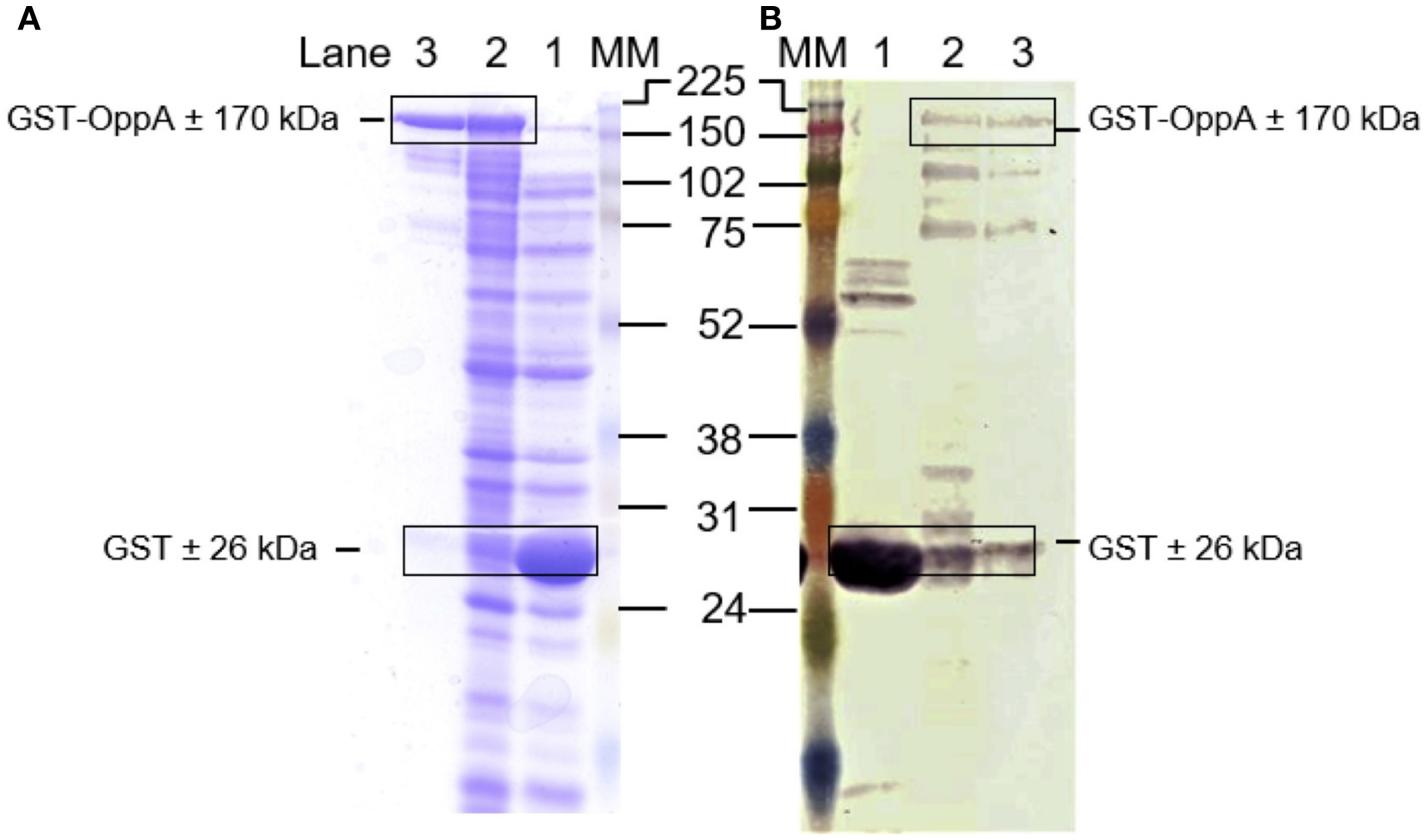
Expression of recombinant GST-OppA protein. **(A)** SDS-PAGE analysis of the expressed OppA protein and **(B)** western blot analysis using anti-GST antibody. Lane 1 *E. coli* BL21(DE3)pLysS cells expressing GST-control, lane 2 *E. coli* BL21(DE3)pLysS cells expressing GST-OppA protein, and lane 3 isolated recombinant GST-OppA protein. MM is the GE Healthcare full-range rainbow molecular weight marker, molecular sizes indicated in kDa.

Sub-cloning of the mutated *oppA* gene into the pGEX-4T-1 vector was successful as confirmed by sequencing (Supplementary Figure 2). SDS-PAGE and western blot analyses confirmed that the recombinant OppA protein was expressed successfully as an N-terminal GST-fusion protein with the predicted size of 170 kDa (26 kDa due to the GST tag) (Figures 1A,B). Bradford analysis indicated that the OppA protein was eluted between fraction 7 and 12 with the highest concentration obtained in the 9th fraction.

#### Evaluation of Anti-OppA Antibody Response

Ostriches with more than two missing sampling points were excluded from ELISA and subsequent statistical analysis. There was no non-specific binding of the anti-OppA antibodies in ostrich serum and the plate control samples indicated limited plate to plate variation.

**Figure 2:**
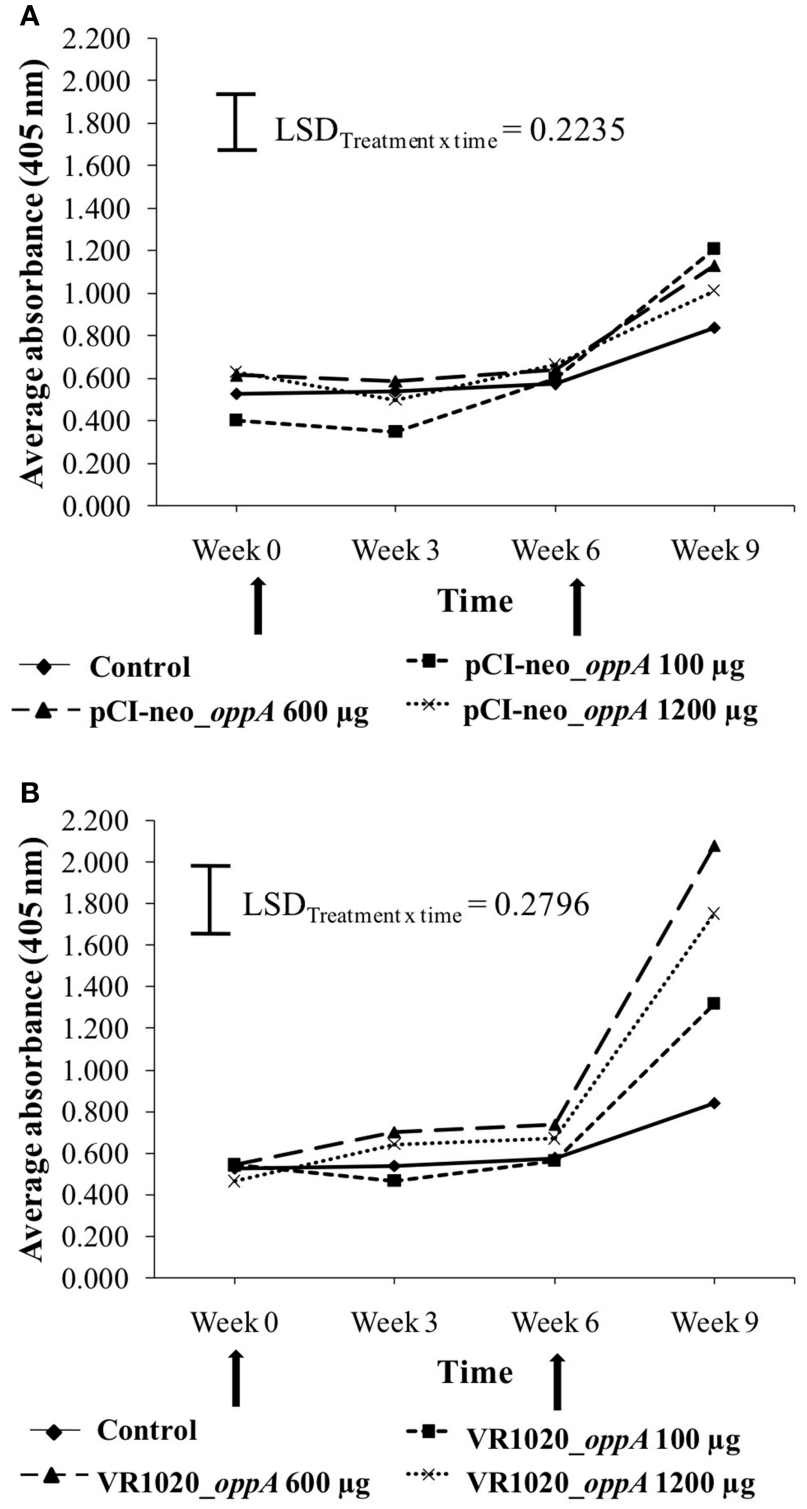
Anti-OppA antibody response elicited in response to vaccination with different doses of the pCI-neo_*oppA* **(A)** and VR1020_*oppA* **(B)** vaccines. The black arrows indicate the time points at which the ostriches were vaccinated. The control group did not receive any vaccine.

The titer values resulting from vaccination with the pCI-neo_*oppA* and VR1020_*oppA* vaccines are shown in Figures 2A,B, respectively. The titer values of the control group, which received no vaccine, did not show significant variation over time. However, there was a slight increase in the average titer at week 9. Vaccination with the pCI-neo_*oppA* vaccine (Figure 2A) resulted in significant treatment x time interactions (*P* = 0.0462), but only the 100 and 600 μg doses resulted in average ELISA titers that differed significantly from the control. This was also only at week 9 after a booster vaccination was administered. The average titers of the different doses were, however, not significantly different from one another. Vaccination with the VR1020_*oppA* vaccine (Figure 2B) resulted in a significant treatment × time interaction (*P* < 0.001) and all three doses resulted in average ELISA titers that differed significantly (LSD = 0.2796) from the control. This was again only at week 9 after a booster vaccination was administered and all the doses differed significantly from one another.

### Monitoring Weight and Mycoplasma Infections

The vaccinated groups as well as the control group gained weight from weeks 0 to 6, but from weeks 6 to 9 there was an average weight loss of 0.05 kg (Table 1). There was, however, no statistically significant difference between the treatment groups and the control group over the 9 weeks period (*P* = 0.9564 for pCI-neo_*oppA* and *P* = 0.5290 for VR1020_*oppA*).

**Table 1:** The average weight gain of ostriches measured during the course of the trial.

**Group**	**Dose**	Group size^*^	**Average weight (kg)**			
			**Week 0**	**Week 3^**#**^**	**Week 6**	**Week 9**
pCI-neo_*oppA*	100 μg	19	26.5	No weight recorded	43.2	44.9
	600 μg	17	26.0		42.7	43.7
	1,200 μg	19	26.8		45.8	45.4
VR1020_*oppA*	100 μg	19	27.5		47.9	47.2
	600 μg	18	26.2		42.7	41.1
	1,200 μg	19	26.0		44.5	42.9
Control	–	17	27.2		44.2	43.8

* *At the start of the trial there was 20 ostriches in each group, during the trial some of the ostriches lost their tags and were excluded from the analysis*.

# *No scale available to record weight*.

The presence of mycoplasma infections was monitored with PCR during the trial (Table 2). Mycoplasma infections could not be detected in any of the groups at weeks 0, 3, and 6. Infections were, however, detected at week 9. At this point the ostriches had been in Oudtshoorn for 36 days, of which 8 days were out of quarantine.

**Table 2:** Summary of mycoplasma infections detected during the vaccine trial using PCR.

**Vaccine**		**Ms01**				**Ms02**				**Ms03**			
	** 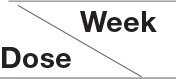 **	**0**	**3**	**6**	**9**	**0**	**3**	**6**	**9**	**0**	**3**	**6**	**9**
pCI-neo_*oppA*	100 μg	–	–	–	1/19 (5.3%)	–	–	–	2/19 (10.5%)	–	–	–	1/19 (5.3%)
	600 μg	–	–	–	–	–	–	–	1/17 (5.9%)	–	–	–	–
	1,200 μg	–	–	–	–	–	–	–	1/19 (5.3%)	–	–	–	1/19 (5.3%)
	Total	**–**	**–**	**–**	1/55 (1.8%)	–	–	–	4/55 (7.2%)	–	–	–	2/55 (3.6%)
VR1020_*oppA*	100 μg	–	–	–	–	–	–	–	2/19 (10.5%)	–	–	–	1/19 (5.3%)
	600 μg	–	–	–	2/18 (11.1%)	–	–	–	4/18 (22.2%)	–	–	–	2/18 (11.1%)
	1,200 μg	–	–	–	–	–	–	–	–	–	–	–	–
	Total	**–**	**–**	**–**	2/57 (3.5%)	–	–	–	6/57 (10.5%)	–	–	–	3/57 (5.2%)
Control		–	–	–	–	–	–	–	–	–	–	–	–

In the groups that received the VR1020_*oppA* vaccine the highest percentage of infections at week 9 was due to Ms02 (10.5%) followed by Ms03 (5.2%) and Ms01 (3.5%) (Table 2). Compared to this, the groups that received the pCI-neo_*oppA* vaccine had fewer mycoplasma infections. Amongst these the highest percentage of infections was again due to Ms02 (7.2%) followed by Ms03 (3.6%) and Ms01 (1.8%). For both vaccine groups, some of the birds were infected by more than one of these *Mycoplasma* species. The only group that had no PCR-detectable mycoplasma infections at week 9 was the group that had received 1,200 μg of the VR1020_*oppA* vaccine and the control group.

## Discussion

In this study, DNA vaccines were developed for ostriches using the *oppA* gene of an ostrich-infecting mycoplasma (Ms03) as vaccine antigen. The expression vectors used (pCI-neo and VR1020), were selected based on DNA vaccine studies in other birds, and on their immunostimulatory characteristics (45–47). After vaccination of ±3-month-old chicks a statistically significant anti-OppA antibody response could not be detected at week 3, although a general trend of increases in the average titer values was observed from week 0 to 3 for groups that received the VR1020_*oppA* vaccines. Based on previous studies using inactivated vaccines in ostriches (42, 48), we expected a primary immune response about 3 weeks after the first vaccination (i.e., between weeks 0 and 6).

After administering a booster vaccination, both DNA vaccines were able to elicit a statistically significant anti-OppA antibody response. As these responses against OppA were significant in comparison to the negative responses following the first vaccinations, this is evidence of a secondary immune response as a result of immune memory. If memory cells are produced after the initial contact with an antigen, subsequent exposure to the same antigen will allow the host to recognize the antigen faster, and with greater magnitude (49, 50).

The responses elicited by the DNA vaccines were, however, dose dependent as well as vector dependent since different results were produced by each vaccine when using the same dose. This highlights a possible role of the vaccine vector in the observed antibody responses. The VR1020_*oppA* vaccine, on average, elicited higher titer values compared to the pCI-neo_*oppA* vaccine, which might be due to the TPA signal sequence of the VR1020 plasmid, which is situated upstream of the *oppA* gene, and assists with protein expression in mammalian cells and export of the protein out of the cell. This would in turn increase the immunogenicity of the antigen (45). In addition to this, sequences in the plasmid backbone have intrinsic immunostimulatory activity which enhance the immune system's response to the expressed protein antigen (51, 52).

Over the whole vaccination trial, the health of the ostriches was monitored by recording weight gain. All groups gained weight up to week 6, but toward week 9 their weight gain decreased. Given that this trend was also observed for the control group ostriches, the weight loss cannot be ascribed to vaccination. The decrease in weight did, however, coincide with the movement of the birds to a new location. Ostriches are known to be severely affected by stress and during this period of adapting to changes in social dynamic and housing environment, reduction in weight gain and even weight loss is normal amongst farmed ostriches (4).

The presence of existing mycoplasma infections at the start of the trial, as well as changes in infection status during the trial, were also monitored by PCR analysis of trachea swabs. Mycoplasma infections could only be detected at week 9, which was after they were moved and exposed to the farm environment in Oudtshoorn for more than 30 days. The absence of detectable mycoplasma infections at the earlier time points may be ascribed to Fraserburg being outside of the commercial ostrich production region. The Oudtshoorn district, on the other hand, has a higher incidence of mycoplasma infections especially during seasonal changes and our trial was conducted during late autumn. Amongst the vaccinated groups, the number of detected Ms03 and Ms01 infections were low compared to Ms02, with only the 1200 μg VR1020_*oppA* and control group having no detectable ostrich-infecting mycoplasmas. In future trials, careful consideration should be given to the timing of the primary and booster vaccination relative to the time of introduction to grow-out conditions.

It is possible that the mycoplasma infections had an influence on the observed antibody responses. In a study done by Yang et al. (26) using recombinant OppA protein as subunit vaccine against *M. catarrhalis* in mice, it was found that the ELISA titer values were already raised at the start of the vaccination trial which they ascribed to the presence of existing systemic antibodies to OppA as a result of existing infections. Despite the possible presence of antibodies due to mycoplasma infections, this did not mask the detection of a dose dependent antibody response to the OppA protein of Ms03 during vaccination.

In conclusion, this is the first study to show that DNA vaccines are able to elicit an antibody response in ostriches against a mycoplasma antigen in spite of ostriches being prone to environmentally induced stress conditions that can suppress immune function. The mycoplasma OppA protein was sufficiently immunogenic to induce an anti-OppA antibody response and this response was firstly, dose dependent and secondly, required a booster vaccination. Further trials are required to determine the extent of protection provided by this mycoplasma antigen relative to the timing of the primary and booster vaccination before introduction to grow-out conditions.

## Ethics Statement

This study (field trial) was carried out in accordance with the recommendations of the South African Department of Agriculture, Forestry and Fisheries (Reference: 12/11/1/1/3) in terms of Section 20 of the Animal Disease Act 1984 (Act No. 35 of 1984). The protocol was approved by the Stellenbosch University Animal Ethics Committee (SU-ACUM 13-00019).

## Author Contributions

MW performed the SDM, prepared the DNA vaccine and expression plasmids, optimized the expression of the recombinant OppA protein, and the initial optimization of the ELISA. HJ further optimized the ELISA and evaluated the anti-OppA antibody responses during the vaccination trial. AO assisted with the vaccine trial. MW wrote the manuscript with support from AB. AB supervised the project with the assistance of DB and AO. AB and DB conceived the original idea. All authors provided critical feedback and helped shape the research, analysis and manuscript.

### Conflict of Interest Statement

The authors declare that the research was conducted in the absence of any commercial or financial relationships that could be construed as a potential conflict of interest.
