# Supplementary material for: DNA Vaccines Against Mycoplasma Elicit Humoral Immune Responses in Ostriches
Source: Front Immunol. 2019 May 14;10:1061. doi: 10.3389/fimmu.2019.01061 (PMC6527592; doi:10.3389/fimmu.2019.01061)
Supplement: Supplementary file 1 [file Data_Sheet_1.docx]

**Supplementary file: DNA vaccines against mycoplasma elicit humoral immune responses in ostriches**

Table of Contents

[Supplementary Procedures 1](#_Toc516234103)

[Site-directed mutagenesis of the *oppA* gene 1](#_Toc516234104)

[Preparation of DNA vaccines 3](#_Toc516234110)

[Expression and purification of the recombinant OppA protein 4](#_Toc516234115)

[Reference list 5](#_Toc516234117)

[Supplementary tables and figures 6](#_Toc516234118)

[Supplementary Table 1 Primers used in this study 6](#_Toc516234119)

[Supplementary Figure 1 Analysis of the integrity of the isolated pCI-neo_](#_Toc516234126)*[oppA](#_Toc516234126)* [and VR1020_](#_Toc516234126)*[oppA](#_Toc516234126)* [DNA vaccine plasmids 8](#_Toc516234126)

Supplementary Figure 2 Sequencing results to verify cloning and SDM of *oppA* gene [9](#_Toc516234125)

# Supplementary Procedures

## Site-directed mutagenesis of the *oppA* gene

*Mycoplasma* species contain two *opp* operons, each with an *oppA* gene [1]. These operons can be divided into three types, of which the Type A is found in all *Mycoplasma* species of the pneumoniae and hominis groups. The immunogenicity and essential nature of the Type A OppA protein has also been confirmed experimentally for *M. hominis* [2, 3]. The Type A *oppA* gene of Ms03 was therefore used in this study. To allow expression of this gene in a eukaryotic host, all mycoplasma tryptophan codons (TGA) were changed to universal tryptophan codons (TGG) using site-directed mutagenesis (SDM). To this end, the *oppA* gene was amplified from Ms03 gDNA using proof-reading Kapa HiFi DNA polymerase (Kapa Biosystems) according to the manufacturer’s instructions. Restriction enzyme cut sites of MluI and AccI were added to the Ms03_P100F and Ms03_P100R primers respectively (Supplementary Table 1) for downstream sub-cloning into the pCI-neo DNA vaccine vector. PCR products were separated and visualised using a 1% (w/v) agarose gel containing 0.5 μg/ml ethidium bromide under UV light. PCR products were purified using the GFX™ PCR DNA and gel band purification kit (GE Healthcare Life Science, UK) according to the manufacturer’s instructions.

For SDM, the purified blunt ended PCR product was cloned into the pGEM^®^-T Easy vector (Promega). To this end, 3' terminal deoxyadenosine residues were added to the blunt ends of the purified PCR product using the A-tailing procedure as described in the pGEM^®^-T Easy system I manual. The A-tailing product was cleaned using a DNA clean and concentrator™-5 kit (ZYMO Research) followed by overnight ligation at 4°C into the pGEM^®^–T Easy vector, according to the manufacturer’s instructions (vector: insert ratio 1:1 and 1:3). The ligation products (pGEM_*oppA*_MluIAccI_) were transformed into *E. coli* JM109 cells and grown on Luria-Bertani (LB) agar plates (15 g/L) containing a final concentration of 100 μg/ml ampicillin (Sigma-Aldrich), 0.16 mM isopropyl β-D-1-thiogalactopyranoside (IPTG, Bioline) and 40 μg/ml X-Gal (Bioline) for 16-18 h at 37°C.

White colonies were screened with colony PCR to confirm the presence of the insert using the pGEM–T Easy_T7 and P100_2R primers (Supplementary Table 1). Each colony PCR reaction contained 1 × reaction buffer, 0.2 mM of each dNTP (Kapa Biosystems), 2 mM MgCl_2_, 1 pmol/ml of each primer and 0.2 units of Super-Therm Taq DNA polymerase (JMR Holdings) in a final volume of 10 μl. Bacteria from a single colony were transferred with a sterilized toothpick into the PCR mixture. PCR conditions were as follows: 25 cycles of 94°C for 30 s, 55°C for 15 s and 72°C for 1 min, followed by a final step at 72°C for 6 min in a Veriti 96 well Thermal Cycler (Applied Biosystems, USA). Positive colonies were inoculated into 5 ml LB medium containing 100 μg/ml ampicillin and grown overnight (16 h) at 37°C on an orbital shaker at 250 rpm. The plasmid DNA (pDNA) was isolated from 2 ml culture with the Invisorb^®^ spin plasmid mini two kit (Invitek GmbH, Germany), according to the manufacturer’s instruction. All pDNA samples were stored at 4°C. Freezer stocks were prepared by diluting 1:1 with 80% glycerol and stored at -80°C.

Accurate insertion of the gene into the vector and absence of point mutations was confirmed by sequencing. Products were analysed by the Central Analytical Facility (CAF), DNA Sequencing Unit of Stellenbosch University using an ABI^®^ 3100 Genetic Analyser (Applied Biosystems, USA). The resulting sequences were aligned and compared to that of the vector and gene sequence of Ms03 *oppA* (GenBank: KM410300.1) using BioEdit v7.0.5.2 [4].

The *oppA* gene contained 16 TGA codons that had to be modified and the positions of these codons (from the translational start) were 12, 15, 1 290, 1 680, 1 762, 2 196, 2 301, 2 550, 2 626, 2 727, 2 775, 3 156, 3 213, 3 225, 3 258 and 3 609. To reduce the number of required SDM steps, a strategy was followed where two primer sets were combined in a single step. Primer sets were combined because they had very similar annealing temperatures but bound to the *oppA* gene at distant positions. In each step, the pGEM_*oppA*_MluIAccI_ vector was amplified with two sets of SDM primers (Supplementary Table 1) with Kapa HiFi DNA polymerase according to the manufacturer’s instructions using 2.25 mM MgCl_2_. The pGEM_*oppA*_MluIAccI_ vector that was not generated by the SDM PCR was removed with DpnI (Promega, USA) treatment according to the manufacturer’s instructions. The enzymes were removed with the DNA clean and concentrator™-5 kit (ZYMO Research, USA) before transforming the plasmids into *E. coli* JM109 cells and growth on LB-agar plates containing a final concentration of 100 μg/ml ampicillin for 16-18 h at 37°C. As before, a colony PCR was used to select positive colonies and the plasmid subsequently isolated and the insert sequenced as before to confirm the success of each SDM step.

## Preparation of DNA vaccines

Two eukaryotic expression vectors were chosen as vaccine vectors namely pCI-neo (Promega) and VR1020 (Vical Inc.). For sub-cloning of the mutated *oppA* gene into pCI-neo, single consecutive digests of the pCI-neo and SDM-pGEM*_oppA*_MluIAccI_ vectors were performed with AccI and MluI (FastDigest, Thermo Scientific) according to the manufacturer’s instructions. The digested products were purified with the Zymo-DNA clean and concentrator™-5 kit (Zymo Research, USA). The linear pCI-neo product was further treated with shrimp alkaline phosphatase (SAP, Promega) according to manufacturer’s instructions and purified as before. Ligation was performed overnight at 4°C with a vector to insert ratio of 1:1 using T4 DNA ligase (Promega) before transforming the plasmids into *E. coli* JM109 cells and grown as before. Positive colonies were identified by colony PCR using T7EEV and P100_2R primers (Supplementary Table 1) and the insert sequenced as described above.

For sub-cloning into VR1020, the *oppA* gene was amplified by PCR from the SDM-pGEM_*oppA*_MluIAccI_ vector using primers with an added BamHI restriction enzyme sites (Supplementary Table 1). The PCR product was then cloned into a pGEM^®^-T Easy vector and pDNA isolated as describe above. The resulting plasmid and the VR1020 vector were digested with BamHI restriction enzyme (Fermentas). Purification, SAP treatment, ligation and transformation were performed as described above except that a different antibiotic was used (kanamycin, 50 μg/ml). Positive colonies were identified using VR1020_F and P100_2R primers (Supplementary Table 1) followed by sequencing of the insert as described above to confirm the correct orientation of the insert.

Starter cultures of *E. coli* JM109 cells containing the pCI-neo_*oppA* or VR1020_*oppA* vaccine plasmid respectively were prepared by inoculating 10 ml 2xYT-medium (16 g/L tryptone, 10 g/L yeast extract and 5 g/L NaCl) with 1 ml overnight culture (prepared from freezer stocks) and overnight cultivation at 37°C on an orbital shaker (150 rpm). Each of the starter cultures were used to inoculate a larger volume of 2xYT-medium (1:20) and cultivated for 16 h at 37°C on an orbital shaker (150 rpm). The pCI-neo_*oppA* cultures contained a final concentration of 100 μg/ml ampicillin whereas the VR1020_*oppA* cultures contained 50 μg/ml kanamycin.

The pCI-neo*_oppA* and VR1020_*oppA* plasmids were purified with an Endotoxin-free plasmid DNA purification kit (NucleoBond^®^ Xtra Midi plus EF, Macherey-Nagel, Germany) according to the manufacturer’s instructions.

## Expression and purification of the recombinant OppA protein

For this purpose, the mutated *oppA* gene had to be sub-cloned into a pGEX-4T-1 vector (GE Healthcare Life Science, UK). To this end, the *oppA* gene was first amplified by PCR from the SDM-pGEM_*oppA*_MluIAccI_ vector using proof-reading Kapa HiFi DNA polymerase and the Ms03_P100BamHI_F and Ms03_P100NotI_R2 primers with BamHI and NotI restriction sites (Supplementary Table 1). The PCR product was next sub-cloned into a pGEM^®^-T Easy vector and the resulting pGEM_*oppA*_BamNot_ plasmid isolated as before. Double digests of the pGEM_*oppA*_BamNot_ and the pGEX-4T-1 vector were performed with BamHI and NotI (FastDigest, Thermo Scientific) and purified as before. The BamHI/NotI digested linear pGEX-4T-1 product was further treated with SAP, before ligation and transformation was performed as before. A control was included which contained digested pGEX-4T-1 without insert. Positive colonies were identified using a colony PCR as described above using primers pGEX_F and P100_2R (Supplementary Table 1) followed by plasmid isolation and sequencing as before.

## Reference list

[1] Wium M, Botes A, Bellstedt DU. The identification of *oppA* gene homologues as part of the oligopeptide transport system in mycoplasmas. Gene. 2015;558:31-40. <http://doi.org/10.1016/j.gene.2014.12.036>.

[2] Henrich B, Hopfe M, Kitzerow A, Hadding U. The adherence-associated lipoprotein P100, encoded by an *opp* operon structure, functions as the oligopeptide-binding domain OppA of a putative oligopeptide transport system in *Mycoplasma hominis*. J Bacteriol. 1999;181:4873-8.

[3] Henrich B, Feldmann R-C, Hadding U. Cytoadhesins of *Mycoplasma hominis*. Infect Immun. 1993;61:2945-51.

[4] Hall TA. BioEdit: A user-friendly biological sequence alignment editor and analysis program for Windows 95/98/NT. Nucleic Acids Symposium Series. 1999;41:95-8.

# Supplementary tables and figures

## Supplementary Table 1 Primers used in this study^1^

| **Primer name** | **Sequence*** | **Anneal. Temp** | **Comment** |
| --- | --- | --- | --- |
| Ms03_P100F^2^ | 5'-ACGCGT**ATG**AAAAAATGATGATTATT-3' | 55 | For cloning into pCI-neo (5' MluI restriction enzyme site) |
| Ms03_P100R^2^ | 5'-GTCGAC**CTA**TTTAGGTCTTACACCGT-3' |  | For cloning into pCI-neo (5' AccI restriction enzyme site) |
| Ms03_P100BamHI_F^3, 4^ | 5'-GGATCC**ATG**AAAAAATGGTGGTTAT-3' | 60 | For cloning into VR1020 and pGEX-4T-1 (5' BamIH restriction enzyme site) |
| Ms03_P100BamHI_R^3^ | 5'-GGATCC**CTA**TTTAGGTCTTACACCG-3' |  | For cloning into VR1020 and pGEX-4T-1 (5' BamIH restriction enzyme site) |
| Ms03_P100NotI_R2^4^ | 5'-ATGCGGCCGC**CTA**TTTAGGTCTTACAC-3' | 55 | For cloning into pGEX-4T-1 (5' NotI restriction enzyme site) |
| T7_R | 5'-GCTGTAATTTGGGCATTTTCTTG-3' | 74 | Internal *oppA* primers, position 592-615 |
| P100_1F | 5'-CATTAATTTAGCTTTATTAAAAGAT-3' | 64 | Internal *oppA* primers, position 549-573 |
| P100_1R | 5'-GAAACAAATGAAATTGAAACAGTAT-3' | 68 | Internal *oppA* primers, position 1 220-1 244 |
| P100_2F | 5'-GACAACACTGTAAGTTTTGGAAATC-3' | 70 | Internal *oppA* primers, position 1 177-1 201 |
| P100_2R^5, 6, 7, 8, 9^ | 5'-ATTTTTCTGGTTTTAATAAGTCATC-3' | 67 | Internal *oppA* primers, position 1 906-1 930 |
| P100_3F | 5'-CCCTAGAGCAAACTATGGAAATAAA-3' | 72 | Internal *oppA* primers, position 1 851-1 875 |
| P100_3R | 5'-CAGAAGAAGGTATTTACTAATGTGT-3' | 65 | Internal *oppA* primers, position 2 525-2 549 |
| P100_4F | 5'-AGAGATGCTGTAAATAAAGATCCTG-3' | 69 | Internal *oppA* primers, position 2 461-2 485 |
| P100_4R | 5'-TGTTCAGTAGTTGGTTTAACAAAGT-3' | 68 | Internal *oppA* primers, position 3 185-3 209 |
| SP6_F | 5'-GCTTTAGATTTAGTAATTGCTGCTT-3' | 70 | Internal *oppA* primers, position 3 130-3 154 |
| pGEM-T Easy_T7^5, 6^ | 5'-TAATACGACTCACTATAGGG-3' | | pGEM^®^-T Easy vector |
| pGEM-T Easy_SP6 | 5'-ATTTAGGTGACACTATAGAA-3' | |  |
| pGEX_F^6, 9^ | 5'-GGGCTGGCAAGCCACGTTTGGTG-3' | | pGEX-4T-1 vector |
| pGEX_R | 5'-CCGGGAGCTGCATGTGTCAGAGG-3' | |  |
| pCI-neo_T7EEV^6,7^ | 5'-AAGGCTAGAGTACTTAATACGA-3' | | pCI-neo vector |
| pCI-neo_T3 | 5'-AATTAACCCTCACTAAAGGG-3' | |  |
| VR1020_F^6, 8^ | 5'-CGTCGACAGAGCTGAGATCCTACAG-3' | | VR1020 vector |
| VR1020_R | 5'-GACACCTACTCAGACAATGCGATGC-3' | |  |
| Site1&2_F | 5'-ACGCGT**ATG**AAAAAATG**G**TG**G**TTATTACCAGTAGCTAGTACA-3' | 66^#^ | SDM reaction 1, mutate positions 12 &15 |
| Site1&2_R | 5'-TGTACTAGCTACTGGTAATAACC**A**CC**A**TTTTTT**CAT**ACGCGT-3' |  |  |
| Site10_F | 5'-TTTTAACAGCAGCTATTGACTG**G**AACTCAATCGCTTCAATT-3' |  | SDM reaction 1, mutate position 2 727 |
| Site10_R | 5'-AATTGAAGCGATTGAGTTCC**A**GTCAATAGCTGCTGTTAAAA-3' |  |  |
| Site9_F | 5'-TGATGAATATGCTTACACAATGTG**G**GGAATGTCAGCAGCAGA-3' | 68^#^ | SDM reaction 2, mutate position 2 626 |
| Site9_R | 5'-TCTGCTGCTGACATTCCCC**A**CATTGTGTAAGCATATTCATCA-3' |  |  |
| Site13&14_F | 5'-AACCAACTACTGAACAATG**G**AGAAATTACTG**G**ACAGGAACTTC TCCATT-3' |  | SDM reaction 2, mutate positions 3 213 & 3 225 |
| Site13&14_R | 5'-AATGGAGAAGTTCCTGTCC**A**GTAATTTCTCC**A**TTGTTCAGTAG TTGGTT-3' |  |  |

**Supplementary Table 1** (Continues)

| Site3_F | 5'-ACTTAAAGCATCTGATAAGTG**G**GAATTAAATGAAAACG-3' | 61^#^ | SDM reaction 3, mutate position 1 290 | |
| --- | --- | --- | --- | --- |
| Site3_R | 5'-CGTTTTCATTTAATTCCC**A**CTTATCAGATGCTTTAAGT-3' |  |  |  |
| Site7_F | 5'-ACAGTAATTAATAAGAATTACTG**G**GACACAGAATATGTTAAT-3' |  | SDM reaction 3, mutate position 2 301 | |
| Site7_R | 5'-ATTAACATATTCTGTGTCCC**A**GTAATTCTTATTAATTACTGT-3' |  |  |  |
| Site4_F | 5'-GAGTTAGACCAGGTCATTTCTG**G**ACCGATGCTAAAGGAAA-3' | 65^#^ | SDM reaction 4, mutate position 1 680 | |
| Site4_R | 5'-TTTCCTTTAGCATCGGTCC**A**GAAATGACCTGGTCTAACTC-3' |  |  |  |
| Site12_F | 5'-TTAGATTTAGTAATTGCTGCTTG**G**AACGGATTAGATCCAAGA-3' |  | SDM reaction 4, mutate position 3 156 | |
| Site12_R | 5'-TCTTGGATCTAATCCGTTCC**A**AGCAGCAATTACTAAATCTAA-3' |  |  |  |
| Site5_F | 5'-GATTATTAAGAACACAAATGTG**G**GACACACCTTATAGGCTA-3' | 64^#^ | SDM reaction 5, mutate position 1 762 | |
| Site5_R | 5'-TAGCCTATAAGGTGTGTCCC**A**CATTTGTGTTCTTAATAATC-3' |  |  |  |
| Site8_F | 5'-CACATTAGTAAATACCTTCTTCTG**G**TCAATTCTGCCAAAAGA-3' |  | SDM reaction 5, mutate position 2 550 | |
| Site8_R | 5'-TCTTTTGGCAGAATTGACC**A**GAAGAAGGTATTTACTAATGTG-3' |  |  |  |
| Site6_F | 5'-CTAAATTGAGTGGTATTTACTG**G**TATGGACTTTCAGTTGAT-3' | 62^#^ | SDM reaction 6, mutate position 2 196 | |
| Site6_R | 5'-ATCAACTGAAAGTCCATACC**A**GTAAATACCACTCAATTTAG-3' |  |  |  |
| Site16_F | 5'-TGTATTTAGTTCAAGATTCTG**G**CTAAACTATACAACATCAC-3' |  | SDM reaction 6, mutate position 3 609 | |
| Site16_R | 5'-GTGATGTTGTATAGTTTAGCC**A**GAATCTTGAACTAAATACA-3' |  |  |  |
| Site11_F | 5'-CCTCAACCGGTTAAACCTTG**G**ATTACAGGATTGTCTCCTGAC-3' | 68^#^ | SDM reaction 7, mutate position 2 775 | |
| Site11_R | 5'-GTCAGGAGACAATCCTGTAATCC**A**AGGTTTAACCGGTTGAGG-3' |  |  |  |
| Site15_F | 5'-TCCATTTTCACTAGCAGGTTG**G**GGTTATGACTACGATGGTAT-3' |  | SDM reaction 7, mutate position 3 258 | |
| Site15_R | 5'-ATACCATCGTAGTCATAACCCC**A**ACCTGCTAGTGAAAATGGA-3' |  |  |  |
| 1 All primers were synthesized and purified by Integrated DNA Technologies (IDT, USA). Restriction enzyme sites, included for cloning purposes, are underlined, the start and termination codons are indicated in bold and the nucleotides changed by site-directed mutagenesis are bold and underlined.  2 The *oppA* gene was amplified from Ms03 gDNA using this primer set. Primer sequences include restriction enzyme cut sites of MluI and AccI respectively.  3 The *oppA* gene was amplified with this primer set from the SDM-pGEM_*oppA*MluIAccI plasmid using proof-reading Kapa HiFi DNA polymerase (Kapa Biosystems) according to the manufacturer’s instructions. Primer sequences include restriction enzyme cut site of BamHI for sub-cloning into VR1020.  4 The *oppA* gene was amplified with this primer set from the SDM-pGEM_*oppA*MluIAccI plasmid using proof-reading Kapa HiFi DNA polymerase (Kapa Biosystems) according to the manufacturer’s instructions. Primer sequences include restriction enzyme cut site of BamHI and NotI for sub-cloning into pGEX-4T-1.  5 Primers used in the colony PCR to confirm the presence of the *oppA* gene *into* pGEM^®^-T Easy  6 Colony PCR reaction contained 1 × reaction buffer, 0.2 mM of each dNTP (Kapa Biosystems), 2 mM MgCl2, 1 pmol/ml of each primer and 0.2 units of Super-Therm Taq DNA polymerase (JMR Holdings) in a final volume of 10 μl. Bacteria from a single colony were transferred with a sterilized toothpick into the PCR mixture. PCR conditions were as follows: 25 cycles of 94°C for 30 s, 55°C for 15 s and 72°C for 1 min, followed by a final step at 72°C for 6 min in a Veriti 96 well Thermal Cycler (Applied Biosystems, USA).  7 Primers used in the colony PCR to confirm the presence of *oppA* gene into pCI-neo.  8 Primers used in colony PCR to confirm the insert of *oppA* gene into VR1020.  9 Primers used in colony PCR to confirm the insert of *oppA* gene into pGEX-4T-1.  # Annealing temperature for SDM PCR reaction using the four primers. | | | |  |


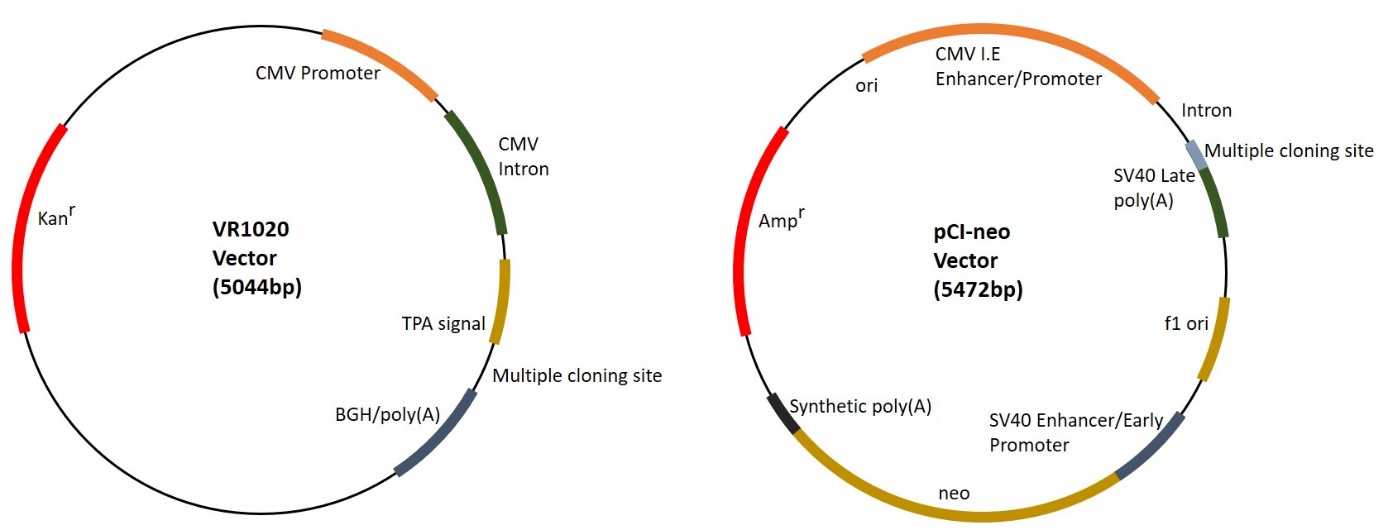


## Supplementary Figure 1 Vector map of the VR1020 and pCI-neo vectors used to develop the vaccines.

## Supplementary Figure 2 Sequencing results to verify cloning and SDM of *oppA* gene

Alignments of the “*Mycoplasma* *nasistruthionis* sp. nov.” str. Ms03 Type A *oppA* gene created using BioEdit. Sequences in the alignment represent the *oppA* gene before SDM, after each round of SDM and after each sub-cloning event.

10 20 30 40 50 60 70 80 90 100 110 120 130 140

....|....|....|....|....|....|....|....|....|....|....|....|....|....|....|....|....|....|....|....|....|....|....|....|....|....|....|....|

***oppA***   **ATGAAAAAATGATGATTATTACCAGTAGCTAGTACAAGTTTAATTCTTCCAGCACTTGCCCTTTCATGTAAAAACACATCAAGCGAAAGAACTTTACACTTTTTATCTGAAAAA**

**pGEM_*oppA* before SDM** **GCGGCCGCGGGAATTCGATTACGCGT..................................................................................................................**

**SDM1 Site 1, 2 & 10**  **GCGGCCGCGGGAATTCGATTACGCGT...........G..G...................................................................................................**

**SDM2 Site 9, 13 & 14** **GCGGCCGCGGGAATTCGATTACGCGT...........G..G...................................................................................................**

**SDM3 Site 3 & 7**  **GCGGCCGCGGGAATTCGATTACGCGT...........G..G...................................................................................................**

**SDM4 Site 4 & 12**  **GCGGCCGCGGGAATTCGATTACGCGT...........G..G...................................................................................................**

**SDM5 Site 5 & 8**  **GCGGCCGCGGGAATTCGATTACGCGT...........G..G...................................................................................................**

**SDM6 Site 6 & 16**  **GCGGCCGCGGGAATTCGATTACGCGT...........G..G...................................................................................................**

**SDM7 Site 11 & 15**  **GCGGCCGCGGGAATTCGATTACGCGT...........G..G...................................................................................................**

**pCI-neo_*oppA***  **TAGGCTAGCCTCGAGAATTCACGCGT...........G..G...................................................................................................**

**pGem_*oppA* BamBam**  **GCGGCCGCGGGAATTCGATTGGATCC...........G..G...................................................................................................**

**VR1020_*oppA***  **TCGTTTCGCCCAGCGGTACCGGATCC...........G..G...................................................................................................**

**pGEM_*oppA* BamNot**  **GCGGCCGCGGGAATTCGATTGGATCC...........G..G...................................................................................................**

**pGEX_*oppA***  **AATCGGATCTGGTTCCGCGTGGATCC...........G..G...................................................................................................**

150 160 170 180 190 200 210 220 230 240 250 260 270 280

....|....|....|....|....|....|....|....|....|....|....|....|....|....|....|....|....|....|....|....|....|....|....|....|....|....|....|....|

***oppA***  **CAAGTTGCTGAATATCAAGAAAAAGCTCAAGAATATTCAAAACAAGCATTAGCTTCACTTGCTGAATTTAATAAATACAGCAAACAAAAaCAACAATTAGAACAACAAGCTGATTTATTAATCTTTTCTTTACAAGACTT**

**pGEM_*oppA* before SDM** **............................................................................................................................................**

**SDM1 Site 1, 2 & 10**  **............................................................................................................................................**

**SDM2 Site 9, 13 & 14**  **............................................................................................................................................**

**SDM3 Site 3 & 7**  **............................................................................................................................................**

**SDM4 Site 4 & 12**  **............................................................................................................................................**

**SDM5 Site 5 & 8**  **............................................................................................................................................**

**SDM6 Site 6 & 16**  **............................................................................................................................................**

**SDM7 Site 11 & 15**  **............................................................................................................................................**

**pCI-neo_*oppA***  **............................................................................................................................................**

**pGem_*oppA* BamBam**  **............................................................................................................................................**

**VR1020_*oppA***  **............................................................................................................................................**

**pGEM_*oppA* BamNot**  **............................................................................................................................................**

**pGEX_*oppA***  **............................................................................................................................................**

**Key**

Start codon

Stop codon

SDM site

BamIH restriction enzyme site

MluI restriction enzyme site

AccI restriction enzyme site

NotI restriction enzyme site

290 300 310 320 330 340 350 360 370 380 390 400 410 420

....|....|....|....|....|....|....|....|....|....|....|....|....|....|....|....|....|....|....|....|....|....|....|....|....|....|....|....|

***oppA***  **AAATTCAGACTTATTAGCTGCTAAAACAAAaTTATTTAACTTACAAGCTAAAAAAGAATTATTATCTTTAGCCCTTGCTTCTAGTGATTTTAGTCAATTACAAGACAAAGTTAAACAATATTCAAATTCAGAATTTAAAA**

**pGEM_*oppA* before SDM** **............................................................................................................................................**

**SDM1 Site 1, 2 & 10**  **............................................................................................................................................**

**SDM2 Site 9, 13 & 14**  **............................................................................................................................................**

**SDM3 Site 3 & 7**  **............................................................................................................................................**

**SDM4 Site 4 & 12**  **............................................................................................................................................**

**SDM5 Site 5 & 8**  **............................................................................................................................................**

**SDM6 Site 6 & 16**  **............................................................................................................................................**

**SDM7 Site 11 & 15**  **............................................................................................................................................**

**pCI-neo_*oppA***  **............................................................................................................................................**

**pGem_*oppA* BamBam**  **............................................................................................................................................**

**VR1020_*oppA***  **............................................................................................................................................**

**pGEM_*oppA* BamNot**  **............................................................................................................................................**

**pGEX_*oppA***  **............................................................................................................................................**

430 440 450 460 470 480 490 500 510 520 530 540 550 560

....|....|....|....|....|....|....|....|....|....|....|....|....|....|....|....|....|....|....|....|....|....|....|....|....|....|....|....|

***oppA***  **TCCAAGATTTTTTAAATACTGAATTTAATAATTATTTAAATAAATCATATGGTGATGAACAAAACCACTTACCAACACTAAAACAACTAACAGACAGAGCTAAAAGTTTAGAAAGTAATTTAAACACACTAAAACAAACA**

**pGEM_*oppA* before SDM** **............................................................................................................................................**

**SDM1 Site 1, 2 & 10**  **............................................................................................................................................**

**SDM2 Site 9, 13 & 14**  **............................................................................................................................................**

**SDM3 Site 3 & 7**  **............................................................................................................................................**

**SDM4 Site 4 & 12**  **............................................................................................................................................**

**SDM5 Site 5 & 8**  **............................................................................................................................................**

**SDM6 Site 6 & 16**  **............................................................................................................................................**

**SDM7 Site 11 & 15**  **............................................................................................................................................**

**pCI-neo_*oppA***  **............................................................................................................................................**

**pGem_*oppA* BamBam**  **............................................................................................................................................**

**VR1020_*oppA***  **............................................................................................................................................**

**pGEM_*oppA* BamNot**  **............................................................................................................................................**

**pGEX_*oppA***  **............................................................................................................................................**

570 580 590 600 610 620 630 640 650 660 670 680 690 700

....|....|....|....|....|....|....|....|....|....|....|....|....|....|....|....|....|....|....|....|....|....|....|....|....|....|....|....|

***oppA***  **TTAGATAATAAAAACATTAATTTAGCTTTATTAAAAGATAAAGCAAATAAAACAGAGCAAGAAAATGCCCAAATTACAGCGCTAGAAGCTGAAATTCAAGCATCAAAAGAACAACAAACAACAATTTCACAAGAATTAGA**

**pGEM_*oppA* before SDM** **............................................................................................................................................**

**SDM1 Site 1, 2 & 10**  **............................................................................................................................................**

**SDM2 Site 9, 13 & 14**  **............................................................................................................................................**

**SDM3 Site 3 & 7**  **............................................................................................................................................**

**SDM4 Site 4 & 12**  **............................................................................................................................................**

**SDM5 Site 5 & 8**  **............................................................................................................................................**

**SDM6 Site 6 & 16**  **............................................................................................................................................**

**SDM7 Site 11 & 15**  **............................................................................................................................................**

**pCI-neo_*oppA***  **............................................................................................................................................**

**pGem_*oppA* BamBam**  **............................................................................................................................................**

**VR1020_*oppA***  **............................................................................................................................................**

**pGEM_*oppA* BamNot**  **............................................................................................................................................**

**pGEX_*oppA***  **............................................................................................................................................**

710 720 730 740 750 760 770 780 790 800 810 820 830 840

....|....|....|....|....|....|....|....|....|....|....|....|....|....|....|....|....|....|....|....|....|....|....|....|....|....|....|....|

***oppA***  **TCAAAATTCTGAAACAATGGCTACAGTTCAGAATCATATTTACCAAGATTTAAAACAACAAGCAAATGATCAAAGTATGTTAGATGCCCAAATTGCACAACAAGAGCAATCAAATCAAGAAGTTCAACAACAAATCAACA**

**pGEM_*oppA* before SDM** **............................................................................................................................................**

**SDM1 Site 1, 2 & 10**  **............................................................................................................................................**

**SDM2 Site 9, 13 & 14**  **............................................................................................................................................**

**SDM3 Site 3 & 7**  **............................................................................................................................................**

**SDM4 Site 4 & 12**  **............................................................................................................................................**

**SDM5 Site 5 & 8**  **............................................................................................................................................**

**SDM6 Site 6 & 16**  **............................................................................................................................................**

**SDM7 Site 11 & 15**  **............................................................................................................................................**

**pCI-neo_*oppA***  **............................................................................................................................................**

**pGem_*oppA* BamBam**  **............................................................................................................................................**

**VR1020_*oppA***  **............................................................................................................................................**

**pGEM_*oppA* BamNot**  **............................................................................................................................................**

**pGEX_*oppA***  **............................................................................................................................................**

850 860 870 880 890 900 910 920 930 940 950 960 970 980

....|....|....|....|....|....|....|....|....|....|....|....|....|....|....|....|....|....|....|....|....|....|....|....|....|....|....|....|

***oppA***  **CAAAACAACAAGAACTATCAACTAAAAAAGCTGAAATTAACAAACAAATCAGCGACCAAGGCTTAAATGAAAAAATTCAAAGTGCTTTTGATACTTATTCTAAGGCAACTGATGAACAAAGAAAATACAATGGTTTACTA**

**pGEM_*oppA* before SDM** **............................................................................................................................................**

**SDM1 Site 1, 2 & 10**  **............................................................................................................................................**

**SDM2 Site 9, 13 & 14** **............................................................................................................................................**

**SDM3 Site 3 & 7**  **............................................................................................................................................**

**SDM4 Site 4 & 12**  **............................................................................................................................................**

**SDM5 Site 5 & 8**  **............................................................................................................................................**

**SDM6 Site 6 & 16**  **............................................................................................................................................**

**SDM7 Site 11 & 15**  **............................................................................................................................................**

**pCI-neo_*oppA***  **............................................................................................................................................**

**pGem_*oppA* BamBam**  **............................................................................................................................................**

**VR1020_*oppA***  **............................................................................................................................................**

**pGEM_*oppA* BamNot**  **............................................................................................................................................**

**pGEX_*oppA***  **............................................................................................................................................**

990 1000 1010 1020 1030 1040 1050 1060 1070 1080 1090 1100 1110 1120

....|....|....|....|....|....|....|....|....|....|....|....|....|....|....|....|....|....|....|....|....|....|....|....|....|....|....|....|

***oppA***  **GTCCAAAATGACATTTCAGCTTATAGTTATTCAACTAAAGATGCTAATTTTGATGAAACAGGAGCTAATTCAGATCCTAAATATTTATCAAAACAAGAAGTCAATAAAATAGTTTTTCCTAATGACCCTTTTGTAAATTC**

**pGEM_*oppA* before SDM** **............................................................................................................................................**

**SDM1 Site 1, 2 & 10**  **............................................................................................................................................**

**SDM2 Site 9, 13 & 14**  **............................................................................................................................................**

**SDM3 Site 3 & 7**  **............................................................................................................................................**

**SDM4 Site 4 & 12**  **............................................................................................................................................**

**SDM5 Site 5 & 8**  **............................................................................................................................................**

**SDM6 Site 6 & 16**  **............................................................................................................................................**

**SDM7 Site 11 & 15**  **............................................................................................................................................**

**pCI-neo_*oppA***  **............................................................................................................................................**

**pGem_*oppA* BamBam**  **............................................................................................................................................**

**VR1020_*oppA***  **............................................................................................................................................**

**pGEM_*oppA* BamNot**  **............................................................................................................................................**

**pGEX_*oppA***  **............................................................................................................................................**

1130 1140 1150 1160 1170 1180 1190 1200 1210 1220 1230 1240 1250 1260

....|....|....|....|....|....|....|....|....|....|....|....|....|....|....|....|....|....|....|....|....|....|....|....|....|....|....|....|

***oppA***  **ACCAGTTAGTGATAGTTTTGCTAAAAACGGTGTTTTCCAAATTGATACAAACTCACAATATTCACCTGGTTATGCTCCATTTGACAACACTGTAAGTTTTGGAAATCGTCAAGCTAATATTTCAGATACTGTTTCAATTT**

**pGEM_*oppA* before SDM** **............................................................................................................................................**

**SDM1 Site 1, 2 & 10**  **............................................................................................................................................**

**SDM2 Site 9, 13 & 14**  **............................................................................................................................................**

**SDM3 Site 3 & 7**  **............................................................................................................................................**

**SDM4 Site 4 & 12**  **............................................................................................................................................**

**SDM5 Site 5 & 8**  **............................................................................................................................................**

**SDM6 Site 6 & 16**  **............................................................................................................................................**

**SDM7 Site 11 & 15**  **............................................................................................................................................**

**pCI-neo_*oppA***  **............................................................................................................................................**

**pGem_*oppA* BamBam**  **............................................................................................................................................**

**VR1020_*oppA***  **............................................................................................................................................**

**pGEM_*oppA* BamNot**  **............................................................................................................................................**

**pGEX_*oppA***  **............................................................................................................................................**

1270 1280 1290 1300 1310 1320 1330 1340 1350 1360 1370 1380 1390 1400

....|....|....|....|....|....|....|....|....|....|....|....|....|....|....|....|....|....|....|....|....|....|....|....|....|....|....|....|

***oppA***  **CATTTGTTTCAGCTGAAAGAATAGGAAAAACAAAACTTAAAGCATCTGATAAGTGAGAATTAAATGAAAACGGTGTTCCTGTTAAAGTAAAAGTTGAAAACATTATTTCACCAACTGTTTTAAGATACAAATTAGAACTA**

**pGEM_*oppA* before SDM** **............................................................................................................................................**

**SDM1 Site 1, 2 & 10**  **............................................................................................................................................**

**SDM2 Site 9, 13 & 14**  **............................................................................................................................................**

**SDM3 Site 3 & 7**  **.......................................................G....................................................................................**

**SDM4 Site 4 & 12**  **.......................................................G....................................................................................**

**SDM5 Site 5 & 8**  **.......................................................G....................................................................................**

**SDM6 Site 6 & 16**  **.......................................................G....................................................................................**

**SDM7 Site 11 & 15**  **.......................................................G....................................................................................**

**pCI-neo_*oppA***  **.......................................................G....................................................................................**

**pGem_*oppA* BamBam**  **.......................................................G....................................................................................**

**VR1020_*oppA***  **.......................................................G....................................................................................**

**pGEM_*oppA* BamNot**  **.......................................................G....................................................................................**

**pGEX_*oppA***  **.......................................................G....................................................................................**

1410 1420 1430 1440 1450 1460 1470 1480 1490 1500 1510 1520 1530 540

....|....|....|....|....|....|....|....|....|....|....|....|....|....|....|....|....|....|....|....|....|....|....|....|....|....|....|....|

***oppA***  **GCAGACGCAATTATTTTAAAAGTGCCTAATGAAAGCGGACAATTAGTAGAAATGGTTTTTGATTCAGATGATGCAGGATTAATTCCGGCACCAACTGAAGTTGTAGTTGAAGATAAATTAGACGAAAACGGAAACCCGGT**

**pGEM_*oppA* before SDM** **............................................................................................................................................**

**SDM1 Site 1, 2 & 10**  **............................................................................................................................................**

**SDM2 Site 9, 13 & 14**  **............................................................................................................................................**

**SDM3 Site 3 & 7**  **............................................................................................................................................**

**SDM4 Site 4 & 12**  **............................................................................................................................................**

**SDM5 Site 5 & 8**  **............................................................................................................................................**

**SDM6 Site 6 & 16**  **............................................................................................................................................**

**SDM7 Site 11 & 15**  **............................................................................................................................................**

**pCI-neo_*oppA***  **............................................................................................................................................**

**pGem_*oppA* BamBam**  **............................................................................................................................................**

**VR1020_*oppA***  **............................................................................................................................................**

**pGEM_*oppA* BamNot**  **............................................................................................................................................**

**pGEX_*oppA***  **............................................................................................................................................**

1550 1560 1570 1580 1590 1600 1610 1620 1630 1640 1650 1660 1670 1680

....|....|....|....|....|....|....|....|....|....|....|....|....|....|....|....|....|....|....|....|....|....|....|....|....|....|....|....|

***oppA***  **ACTTGATGAAAACGGTCAAGTTGTTAAAGAAACAAACAAATACTTTGCCAGTGCTGAAGTTAGAAGATTTTCATCAAATCCTAAATCAATTAACTCACAGCATTTCTTTGATGTTTTAAATAAATCAACTGAACTTAAAT**

**pGEM_*oppA* before SDM** **............................................................................................................................................**

**SDM1 Site 1, 2 & 10**  **............................................................................................................................................**

**SDM2 Site 9, 13 & 14**  **............................................................................................................................................**

**SDM3 Site 3 & 7**  **............................................................................................................................................**

**SDM4 Site 4 & 12**  **............................................................................................................................................**

**SDM5 Site 5 & 8**  **............................................................................................................................................**

**SDM6 Site 6 & 16**  **............................................................................................................................................**

**SDM7 Site 11 & 15**  **............................................................................................................................................**

**pCI-neo_*oppA***  **............................................................................................................................................**

**pGem_*oppA* BamBam**  **............................................................................................................................................**

**VR1020_*oppA***  **............................................................................................................................................**

**pGEM_*oppA* BamNot**  **............................................................................................................................................**

**pGEX_*oppA***  **............................................................................................................................................**

1690 1700 1710 1720 1730 1740 1750 1760 1770 1780 1790 1800 1810 1820

....|....|....|....|....|....|....|....|....|....|....|....|....|....|....|....|....|....|....|....|....|....|....|....|....|....|....|....|

***oppA***  **TCAGAGTTAGACCAGGTCATTTCTGAACCGATGCTAAAGGAAATCGAACAAAATATCCAATAGTAGCAAAAGACTTCTATCTTGGATTATTAAGAACACAAATGTGAGACACACCTTATAGGCTATCACATGGTGGTTCA**

**pGEM_*oppA* before SDM** **............................................................................................................................................**

**SDM1 Site 1, 2 & 10**  **............................................................................................................................................**

**SDM2 Site 9, 13 & 14** **............................................................................................................................................**

**SDM3 Site 3 & 7**  **............................................................................................................................................**

**SDM4 Site 4 & 12**  **.........................G..................................................................................................................**

**SDM5 Site 5 & 8**  **.........................G................................................................................G.................................**

**SDM6 Site 6 & 16**  **.........................G................................................................................G.................................**

**SDM7 Site 11 & 15**  **.........................G................................................................................G.................................**

**pCI-neo_*oppA***  **.........................G................................................................................G.................................**

**pGem_*oppA* BamBam**  **.........................G................................................................................G.................................**

**VR1020_*oppA***  **.........................G................................................................................G.................................**

**pGEM_*oppA* BamNot**  **.........................G................................................................................G.................................**

**pGEX_*oppA***  **.........................G................................................................................G.................................**

1830 1840 1850 1860 1870 1880 1890 1900 1910 1920 1930 1940 1950 1960

....|....|....|....|....|....|....|....|....|....|....|....|....|....|....|....|....|....|....|....|....|....|....|....|....|....|....|....|

***oppA***  **AGGGAAACAGATAATGATGTTAGAAGTATGCTAATTAATCCTGGGAGATTCTTAGACCCTAGAGCAAACTATGGAAATAAATATTTATTTTCATTATTTAATGTAAGTTTTGATGACTTATTAAAACCAGAAAAATCATT**

**pGEM_*oppA* before SDM** **............................................................................................................................................**

**SDM1 Site 1, 2 & 10**  **............................................................................................................................................**

**SDM2 Site 9, 13 & 14**  **............................................................................................................................................**

**SDM3 Site 3 & 7**  **............................................................................................................................................**

**SDM4 Site 4 & 12**  **............................................................................................................................................**

**SDM5 Site 5 & 8**  **............................................................................................................................................**

**SDM6 Site 6 & 16**  **............................................................................................................................................**

**SDM7 Site 11 & 15**  **............................................................................................................................................**

**pCI-neo_*oppA***  **............................................................................................................................................**

**pGem_*oppA* BamBam**  **............................................................................................................................................**

**VR1020_*oppA***  **............................................................................................................................................**

**pGEM_*oppA* BamNot**  **............................................................................................................................................**

**pGEX_*oppA***  **............................................................................................................................................**

1970 1980 1990 2000 2010 2020 2030 2040 2050 2060 2070 2080 2090 2100

....|....|....|....|....|....|....|....|....|....|....|....|....|....|....|....|....|....|....|....|....|....|....|....|....|....|....|....|

***oppA***  **ATCAGAAGATGACAATTACACATACTTCACAATTCACAGAGAGGATGAAAGTGTTCCAATTACACAATTTGATAAAGTCCTTGAAAaTGTCTCTTCTTCATATGAATTTATTCCGGCACCTTCTGAATATATCATCAACA**

**pGEM_*oppA* before SDM** **............................................................................................................................................**

**SDM1 Site 1, 2 & 10**  **............................................................................................................................................**

**SDM2 Site 9, 13 & 14**  **............................................................................................................................................**

**SDM3 Site 3 & 7**  **............................................................................................................................................**

**SDM4 Site 4 & 12**  **............................................................................................................................................**

**SDM5 Site 5 & 8**  **............................................................................................................................................**

**SDM6 Site 6 & 16**  **............................................................................................................................................**

**SDM7 Site 11 & 15**  **............................................................................................................................................**

**pCI-neo_*oppA***  **............................................................................................................................................**

**pGem_*oppA* BamBam**  **............................................................................................................................................**

**VR1020_*oppA***  **............................................................................................................................................**

**pGEM_*oppA* BamNot**  **............................................................................................................................................**

**pGEX_*oppA***  **............................................................................................................................................**

2110 2120 2130 2140 2150 2160 2170 2180 2190 2200 2210 2220 2230 2240

....|....|....|....|....|....|....|....|....|....|....|....|....|....|....|....|....|....|....|....|....|....|....|....|....|....|....|....|

***oppA***  **CTTCAAAAGCAAATGCTGAAACAGTAATTTCACAAAAAGATTTAAGTGATGCTCAACTCAAAGCAATCAAATCATCAATCCAAAATGCTAAAGGATTGGCTAAATTGAGTGGTATTTACTGATATGGACTTTCAGTTGAT**

**pGEM_*oppA* before SDM** **............................................................................................................................................**

**SDM1 Site 1, 2 & 10**  **............................................................................................................................................**

**SDM2 Site 9, 13 & 14**  **............................................................................................................................................**

**SDM3 Site 3 & 7**  **............................................................................................................................................**

**SDM4 Site 4 & 12**  **............................................................................................................................................**

**SDM5 Site 5 & 8**  **............................................................................................................................................**

**SDM6 Site 6 & 16**  **.........................................................................................................................G..................**

**SDM7 Site 11 & 15**  **.........................................................................................................................G..................**

**pCI-neo_*oppA***  **.........................................................................................................................G..................**

**pGem_*oppA* BamBam**  **.........................................................................................................................G..................**

**VR1020_*oppA***  **.........................................................................................................................G..................**

**pGEM_*oppA* BamNot**  **.........................................................................................................................G..................**

**pGEX_*oppA***  **.........................................................................................................................G..................**

2250 2260 2270 2280 2290 2300 2310 2320 2330 2340 2350 2360 2370 2380

....|....|....|....|....|....|....|....|....|....|....|....|....|....|....|....|....|....|....|....|....|....|....|....|....|....|....|....|

***oppA***  **GATACTTTATATTCAGGTAAATACATCGGAGAAGACTTTAATCCAGACACATTAACAATTTCAACAGTAATTAATAAGAATTACTGAGACACAGAATATGTTAATGATTTAACAACAATTAAAAAATtCTCAAACAAAtA**

**pGEM_*oppA* before SDM** **............................................................................................................................................**

**SDM1 Site 1, 2 & 10**  **............................................................................................................................................**

**SDM2 Site 9, 13 & 14** **............................................................................................................................................**

**SDM3 Site 3 & 7**  **......................................................................................G.....................................................**

**SDM4 Site 4 & 12**  **......................................................................................G.....................................................**

**SDM5 Site 5 & 8**  **......................................................................................G.....................................................**

**SDM6 Site 6 & 16**  **......................................................................................G.....................................................**

**SDM7 Site 11 & 15**  **......................................................................................G.....................................................**

**pCI-neo_*oppA***  **......................................................................................G.....................................................**

**pGem_*oppA* BamBam**  **......................................................................................G.....................................................**

**VR1020_*oppA***  **......................................................................................G.....................................................**

**pGEM_*oppA* BamNot**  **......................................................................................G.....................................................**

**pGEX_*oppA***  **......................................................................................G.....................................................**

2390 2400 2410 2420 2430 2440 2450 2460 2470 2480 2490 2500 2510 2520

....|....|....|....|....|....|....|....|....|....|....|....|....|....|....|....|....|....|....|....|....|....|....|....|....|....|....|....|

***oppA***  **TGCTTCAGCACCAGTTGAACCAGCTACTTATTCAGaTTTATCATACTTCTCATAtCTTTCGGGACAAAAAGCCACATATCCATTTACAACCTTATCAAAAGCTAATAGAGATGCTGTAAATAAAGATCCTGAAGGATATG**

**pGEM_*oppA* before SDM** **............................................................................................................................................**

**SDM1 Site 1, 2 & 10**  **............................................................................................................................................**

**SDM2 Site 9, 13 & 14**  **............................................................................................................................................**

**SDM3 Site 3 & 7**  **............................................................................................................................................**

**SDM4 Site 4 & 12**  **............................................................................................................................................**

**SDM5 Site 5 & 8**  **............................................................................................................................................**

**SDM6 Site 6 & 16**  **............................................................................................................................................**

**SDM7 Site 11 & 15**  **............................................................................................................................................**

**pCI-neo_*oppA***  **............................................................................................................................................**

**pGem_*oppA* BamBam**  **............................................................................................................................................**

**VR1020_*oppA***  **............................................................................................................................................**

**pGEM_*oppA* BamNot**  **............................................................................................................................................**

**pGEX_*oppA***  **............................................................................................................................................**

2530 2540 2550 2560 2570 2580 2590 2600 2610 2620 2630 2640 2650 2660

....|....|....|....|....|....|....|....|....|....|....|....|....|....|....|....|....|....|....|....|....|....|....|....|....|....|....|....|

***oppA***  **GAATTGCTTATATTCGTGCTTTATCTAAAAACACATTAGTAAATACCTTCTTCTGATCAATTCTGCCAAAAGAAGGTAATTCAGCACCATATTACAATGATGAATATGCTTACACAATGTGAGGAATGTCAGCAGCAGAA**

**pGEM_*oppA* before SDM** **............................................................................................................................................**

**SDM1 Site 1, 2 & 10**  **............................................................................................................................................**

**SDM2 Site 9, 13 & 14**  **.........................................................................................................................G..................**

**SDM3 Site 3 & 7**  **.........................................................................................................................G..................**

**SDM4 Site 4 & 12**  **.........................................................................................................................G..................**

**SDM5 Site 5 & 8**  **.......................................................G.................................................................G..................**

**SDM6 Site 6 & 16**  **.......................................................G.................................................................G..................**

**SDM7 Site 11 & 15**  **.......................................................G.................................................................G..................**

**pCI-neo_*oppA***  **.......................................................G.................................................................G..................**

**pGem_*oppA* BamBam**  **.......................................................G.................................................................G..................**

**VR1020_*oppA***  **.......................................................G.................................................................G..................**

**pGEM_*oppA* BamNot**  **.......................................................G.................................................................G..................**

**pGEX_*oppA***  **.......................................................G.................................................................G..................**

2670 2680 2690 2700 2710 2720 2730 2740 2750 2760 2770 2780 2790 2800

....|....|....|....|....|....|....|....|....|....|....|....|....|....|....|....|....|....|....|....|....|....|....|....|....|....|....|....|

***oppA***  **TCAATTAGTACAAGTTCAAAAAATGCTATAAGAGAAGCAACCGCAGGAACTGGAGGAGAATTTAGAAGCATTTtAACAGCAGCTATTGACTGAAACTCAATCGCTTCAATTCAGCGTTCACCTCAACCGGTTAAACCTTG**

**pGEM_*oppA* before SDM** **............................................................................................................................................**

**SDM1 Site 1, 2 & 10**  **............................................................................................G...............................................**

**SDM2 Site 9, 13 & 14**  **............................................................................................G...............................................**

**SDM3 Site 3 & 7**  **............................................................................................G...............................................**

**SDM4 Site 4 & 12**  **............................................................................................G...............................................**

**SDM5 Site 5 & 8**  **............................................................................................G...............................................**

**SDM6 Site 6 & 16**  **............................................................................................G...............................................**

**SDM7 Site 11 & 15**  **............................................................................................G...............................................**

**pCI-neo_*oppA***  **............................................................................................G...............................................**

**pGem_*oppA* BamBam**  **............................................................................................G...............................................**

**VR1020_*oppA***  **............................................................................................G...............................................**

**pGEM_*oppA* BamNot**  **............................................................................................G...............................................**

**pGEX_*oppA***  **............................................................................................G...............................................**

2810 2820 2830 2840 2850 2860 2870 2880 2890 2900 2910 2920 2930 2940

....|....|....|....|....|....|....|....|....|....|....|....|....|....|....|....|....|....|....|....|....|....|....|....|....|....|....|....|

***oppA***  **AATTACAGGATTGTCTCCTGACTCTAAAATTAATGAACAAAACGaCACAGAAACAACTGTTCCAAATAACTTAAGAGACAACAACGACTTAATTAATGCGGTTTTTGTTGTTGATTCAGAAACAGGACAAAGAGTTAACT**

**pGEM_*oppA* before SDM** **............................................................................................................................................**

**SDM1 Site 1, 2 & 10**  **............................................................................................................................................**

**SDM2 Site 9, 13 & 14** **............................................................................................................................................**

**SDM3 Site 3 & 7**  **............................................................................................................................................**

**SDM4 Site 4 & 12**  **............................................................................................................................................**

**SDM5 Site 5 & 8**  **............................................................................................................................................**

**SDM6 Site 6 & 16**  **............................................................................................................................................**

**SDM7 Site 11 & 15**  **G...........................................................................................................................................**

**pCI-neo_*oppA***  **G...........................................................................................................................................**

**pGem_*oppA* BamBam**  **G...........................................................................................................................................**

**VR1020_*oppA***  **G...........................................................................................................................................**

**pGEM_*oppA* BamNot**  **G...........................................................................................................................................**

**pGEX_*oppA***  **G...........................................................................................................................................**

2950 2960 2970 2980 2990 3000 3010 3020 3030 3040 3050 3060 3070 3080

....|....|....|....|....|....|....|....|....|....|....|....|....|....|....|....|....|....|....|....|....|....|....|....|....|....|....|....|

***oppA***  **TTGGTGAACTGGGTTCATTAATTAAACCAAGTTTTACAAACAATGTTAACGTTTCAGCATCTGATGTTGCTAAATCAGTTGTTTATAGTCAATTACAAGAAAGAATGAAGAATTTATTAGACAGAGTTTATGCTAAGTTC**

**pGEM_*oppA* before SDM** **............................................................................................................................................**

**SDM1 Site 1, 2 & 10**  **............................................................................................................................................**

**SDM2 Site 9, 13 & 14** **............................................................................................................................................**

**SDM3 Site 3 & 7**  **............................................................................................................................................**

**SDM4 Site 4 & 12**  **............................................................................................................................................**

**SDM5 Site 5 & 8**  **............................................................................................................................................**

**SDM6 Site 6 & 16**  **............................................................................................................................................**

**SDM7 Site 11 & 15**  **............................................................................................................................................**

**pCI-neo_*oppA***  **............................................................................................................................................**

**pGem_*oppA* BamBam**  **............................................................................................................................................**

**VR1020_*oppA***  **............................................................................................................................................**

**pGEM_*oppA* BamNot**  **............................................................................................................................................**

**pGEX_*oppA***  **............................................................................................................................................**

3090 3100 3110 3120 3130 3140 3150 3160 3170 3180 3190 3200 3210 3220

....|....|....|....|....|....|....|....|....|....|....|....|....|....|....|....|....|....|....|....|....|....|....|....|....|....|....|....|

***oppA***  **AACATTCCTTCAACTAACAAAATTAGTTTTGATATCTACTACAGATACTTAAACTACCCAGATCCAGTAATTAATGCTTTAGATTTAGTAATTGCTGCTTGAAACGGATTAGATCCAAGAATGAATGTAAACTTTGTTAA**

**pGEM_*oppA* before SDM** **............................................................................................................................................**

**SDM1 Site 1, 2 & 10**  **............................................................................................................................................**

**SDM2 Site 9, 13 & 14**  **............................................................................................................................................**

**SDM3 Site 3 & 7**  **............................................................................................................................................**

**SDM4 Site 4 & 12**  **.....................................................................................................G......................................**

**SDM5 Site 5 & 8**  **.....................................................................................................G......................................**

**SDM6 Site 6 & 16**  **.....................................................................................................G......................................**

**SDM7 Site 11 & 15**  **.....................................................................................................G......................................**

**pCI-neo_*oppA***  **.....................................................................................................G......................................**

**pGem_*oppA* BamBam**  **.....................................................................................................G......................................**

**VR1020_*oppA***  **.....................................................................................................G......................................**

**pGEM_*oppA* BamNot**  **.....................................................................................................G......................................**

**pGEX_*oppA***  **.....................................................................................................G......................................**

3230 3240 3250 3260 3270 3280 3290 3300 3310 3320 3330 3340 3350 3360

....|....|....|....|....|....|....|....|....|....|....|....|....|....|....|....|....|....|....|....|....|....|....|....|....|....|....|....|

***oppA***  **ACCAACTACTGAACAATGAAGAAATTACTGAACAGGAACTTCTCCATTTTCACTAGCAGGTTGAGGTTATGACTACGATGGTATCGGTTCAGGAATTGATGgATATTCATTAAATGCCAAAATCATTCCAACATtATTtG**

**pGEM_*oppA* before SDM** **............................................................................................................................................**

**SDM1 Site 1, 2 & 10**  **............................................................................................................................................**

**SDM2 Site 9, 13 & 14** **..................G...........G.............................................................................................................**

**SDM3 Site 3 & 7**  **..................G...........G.............................................................................................................**

**SDM4 Site 4 & 12**  **..................G...........G.............................................................................................................**

**SDM5 Site 5 & 8**  **..................G...........G.............................................................................................................**

**SDM6 Site 6 & 16**  **..................G...........G.............................................................................................................**

**SDM7 Site 11 & 15**  **..................G...........G................................G............................................................................**

**pCI-neo_*oppA***  **..................G...........G................................G............................................................................**

**pGem_*oppA* BamBam**  **..................G...........G................................G............................................................................**

**VR1020_*oppA***  **..................G...........G................................G............................................................................**

**pGEM_*oppA* BamNot**  **..................G...........G................................G............................................................................**

**pGEX_*oppA***  **..................G...........G................................G............................................................................**

3370 3380 3390 3400 3410 3420 3430 3440 3450 3460 3470 3480 3490 3500

....|....|....|....|....|....|....|....|....|....|....|....|....|....|....|....|....|....|....|....|....|....|....|....|....|....|....|....|

***oppA***  **CCATAGTTGCTGATCCAGAATATGCAGCTAAAATGCAAAATCTTTATCCTCAACTATACAAAGCTGCTCAATATCTAAAaGAGTTTGTCCAAATGAATCGTTTCAGACCTTCAATTTCTCTTGATGACTTTACAAATAAA**

**pGEM_*oppA* before SDM** **............................................................................................................................................**

**SDM1 Site 1, 2 & 10**  **............................................................................................................................................**

**SDM2 Site 9, 13 & 14** **............................................................................................................................................**

**SDM3 Site 3 & 7**  **............................................................................................................................................**

**SDM4 Site 4 & 12**  **............................................................................................................................................**

**SDM5 Site 5 & 8**  **............................................................................................................................................**

**SDM6 Site 6 & 16**  **............................................................................................................................................**

**SDM7 Site 11 & 15**  **............................................................................................................................................**

**pCI-neo_*oppA***  **............................................................................................................................................**

**pGem_*oppA* BamBam**  **............................................................................................................................................**

**VR1020_*oppA***  **............................................................................................................................................**

**pGEM_*oppA* BamNot**  **............................................................................................................................................**

**pGEX_*oppA***  **............................................................................................................................................**

3510 3520 3530 3540 3550 3560 3570 3580 3590 3600 3610 3620 3630 3640

....|....|....|....|....|....|....|....|....|....|....|....|....|....|....|....|....|....|....|....|....|....|....|....|....|....|....|....|

***oppA***  **TTAACAAATTCAAATGTTCAAGATATAGAaCATTACTTTGGTTCATTCAAATATGAAAACGATGGATTTGTTGAATTATCAGCCGAAGAAGCTTCACAATATGTTGACATTAGTGTATTTAGTTCAAGATTCTGACTAAA**

**pGEM_*oppA* before SDM** **............................................................................................................................................**

**SDM1 Site 1, 2 & 10**  **............................................................................................................................................**

**SDM2 Site 9, 13 & 14**  **............................................................................................................................................**

**SDM3 Site 3 & 7**  **............................................................................................................................................**

**SDM4 Site 4 & 12**  **............................................................................................................................................**

**SDM5 Site 5 & 8**  **............................................................................................................................................**

**SDM6 Site 6 & 16**  **......................................................................................................................................G.....**

**SDM7 Site 11 & 15**  **......................................................................................................................................G.....**

**pCI-neo_*oppA***  **......................................................................................................................................G.....**

**pGem_*oppA* BamBam**  **......................................................................................................................................G.....**

**VR1020_*oppA***  **......................................................................................................................................G.....**

**pGEM_*oppA* BamNot**  **......................................................................................................................................G.....**

**pGEX_*oppA***  **......................................................................................................................................G.....**

3650 3660 3670 3680 3690 3700 3710 3720 3730 3740 3750 3760 3770 3780

....|....|....|....|....|....|....|....|....|....|....|....|....|....|....|....|....|....|....|....|....|....|....|....|....|....|....|....|

***oppA***  **CTATACAACATCACCAGTAATTGACGATCCACAAAATCCAGGTCAAAAGAAAATCATTGATCGTTTAGACTTAGTTGAATTAGCTCAAGAAGTGTCAAACTTAGCTGGCGCTATTCCAGACATTAATTTAGCAGTTTCAA**

**pGEM_*oppA* before SDM** **............................................................................................................................................**

**SDM1 Site 1, 2 & 10**  **............................................................................................................................................**

**SDM2 Site 9, 13 & 14** **............................................................................................................................................**

**SDM3 Site 3 & 7**  **............................................................................................................................................**

**SDM4 Site 4 & 12**  **............................................................................................................................................**

**SDM5 Site 5 & 8**  **............................................................................................................................................**

**SDM6 Site 6 & 16**  **............................................................................................................................................**

**SDM7 Site 11 & 15**  **............................................................................................................................................**

**pCI-neo_*oppA***  **............................................................................................................................................**

**pGem_*oppA* BamBam**  **............................................................................................................................................**

**VR1020_*oppA***  **............................................................................................................................................**

**pGEM_*oppA* BamNot**  **............................................................................................................................................**

**pGEX_*oppA***  **............................................................................................................................................**

3790 3800 3810 3820 3830 3840 3850 3860 3870 3880 3890 3900 3910

....|....|....|....|....|....|....|....|....|....|....|....|....|....|....|....|....|....|....|....|....|....|....|....|....|....|....|....

***oppA***  **CACAAAAATATTCAAAAaCATTAATTAATCCAAATTACATCGTGCCTACAAACTTTTCAGACTATGATGATTTCCAAAGATATAGAACTGTAAACGGTGTAAGACCTAAATAG**

**pGEM_*oppA* before SDM** **.................................................................................................................GTCGACAATCACTAGTGAATTCGCGG**

**SDM1 Site 1, 2 & 10**  **.................................................................................................................GTCGACAATCACTAGTGAATTCGCGG**

**SDM2 Site 9, 13 & 14**  **.................................................................................................................GTCGACAATCACTAGTGAATTCGCGG**

**SDM3 Site 3 & 7**  **.................................................................................................................GTCGACAATCACTAGTGAATTCGCGG**

**SDM4 Site 4 & 12**  **.................................................................................................................GTCGACAATCACTAGTGAATTCGCGG**

**SDM5 Site 5 & 8**  **.................................................................................................................GTCGACAATCACTAGTGAATTCGCGG**

**SDM6 Site 6 & 16**  **.................................................................................................................GTCGACAATCACTAGTGAATTCGCGG**

**SDM7 Site 11 & 15**  **.................................................................................................................GTCGACAATCACTAGTGAATTCGCGG**

**pCI-neo_*oppA***  **.................................................................................................................GTCGACCCCGGGCGGCCGCTTCCCTT**

**pGem_*oppA* BamBam**  **.................................................................................................................GGATCCAATCACTAGTGAATTCGCGG**

**VR1020_*oppA***  **.................................................................................................................GGATCCAGATCTGCTGTGCCTTCTAG**

**pGEM_*oppA* BamNot**  **.................................................................................................................GCGGCCGCATAATCACTAGTGAATTC**

**pGEX_*oppA***  **.................................................................................................................GCGGCCGCATCGTGACTGACTGACGA**

**Key**

Start codon

Stop codon

SDM site

BamIH restriction enzyme site

MluI restriction enzyme site

AccI restriction enzyme site

NotI restriction enzyme site
